# Utility of compartmental models to test the competing hypotheses of pathogen evolution and human intervention

**DOI:** 10.3389/fpubh.2025.1702428

**Published:** 2026-01-13

**Authors:** Barsha Saha, Majid Bani-Yaghoub, Chandranath Podder

**Affiliations:** 1 Division of Computing, Analytics & Mathematics, School of Science and Engineering, University of Missouri-Kansas City, Kansas City, MO, United States; 2 Department of Mathematics, University of Dhaka, Dhaka, Bangladesh

**Keywords:** evolution, host-pathogen interpaly, model-based hypothesis testing, transmissibility, virulence

## Abstract

Compartmental models are essential for studying host-pathogen dynamics, evaluating intervention effectiveness, and predicting infection trends. However, the utility of these models for testing competing hypotheses is often overlooked. To address this, we propose a new model-based hypothesis testing (MBHT) approach, which uses compartmental models to evaluate the hypotheses in epidemiology. In our case, using the COVID-19 pandemic as a case study, we formulate hypotheses of SARS-CoV-2 mutation and construct a transmission model to test them. In addition to analyzing steady-state stability, deriving the basic reproduction number, and identifying a backward bifurcation, the model is fitted to seven peaks of U.S. COVID-19 data, each corresponding to periods of viral mutation and morbidity peaks. The estimated posterior probabilities reveal that Short-term within host selection primarily shaped mutations during the early pandemic stages, followed by immune selection driven by natural and vaccine-induced immunity. In later stages, mutations aligned with vaccination-induced virulence and transmission-virulence correlation, while the declining virulence and immune selection partially explained the final stages of SARS-CoV-2 mutation. In conclusion, model-based hypothesis testing offers a powerful yet underutilized approach to uncovering drivers of viral mutation and gaining deeper insights into pathogen evolution during outbreaks.

## 1 Introduction

### 1.1 Compartmental disease models

Compartmental disease models have been essential public health and epidemiology tools, enabling researchers to examine disease dynamics, evaluate interventions, and understand pathogen evolution (1). These models help researchers and public health officials understand how diseases progress over time and how individuals transition through various states of infection during that disease progression. These transitions typically include compartments such as susceptible (*S*), infectious (*I*), recovered (*R*), and also some others, depending on the complexity of the model (2). The modeling approach provides a structured way to understand disease dynamics, evaluate interventions, and anticipate healthcare needs (1, 2). Table 1 describes some key disease modeling applications in outbreak response and strategic healthcare planning.

**Table 1:** Common applications of infectious disease modeling in public health and epidemiology.

**Application**	**Description**	**References**
Analyze disease dynamics	Tracking transitions between various states, such as susceptible, infected, and recovered, to comprehend how infection spreads or dies out over time.	(3, 4, 11)
Predict outbreaks	By simulating different environmental and social conditions, forecast the strength of outbreaks and estimate the potential scale of spread, peak, and duration of outbreaks	(2, 9–11, 74)
Evaluate interventions	Assess the effectiveness of public health interventions such as vaccination and social distancing to estimate how different strategies can reduce transmission and control outbreaks	(1, 12, 27, 31)
Estimate health resources needed	Determine healthcare resource needs during an outbreak, including staffing, hospital beds, and medical supplies, and estimate the burden on healthcare systems	(1, 14)
Predict long-term infection trends	Investigate the effects of immunity, seasonal patterns, or demographic changes on disease prevalence over time, and identify persistent patterns or emerging threats.	(10, 15, 78)
Eco-evolutionary analysis	Analyze the interactions between pathogen adaptation and host responses, incorporating feedback loops like how pathogens evolve in response to human interventions and environmental pressures.	(16–18)

Compartmental models have been used to analyze disease progression over time, providing insights into mechanisms driving disease spread and control (3, 4). They estimate key epidemiological parameters, such as transmission rates and the numbers of infected and recovered individuals in a population over a given period. By formulating transitions between compartments using delay, ordinary, or partial differential equations (5, 6), these models predict how population states change under single or combined interventions, such as vaccination and social distancing, and can reveal outbreak cycles and epidemic trends. In the basic SIR model, for instance, the flow from susceptible to infectious to recovered captures the dynamics of diseases such as measles (7) or salmonella (8), helping define rates of infection and recovery.

They also allow estimation of outbreak features such as spread, peak, duration, and final size under varying conditions (2, 9–11), and support evaluation of public health strategies including vaccination, quarantine, and social distancing (1, 12, 13). For example, incorporating vaccination rates enables assessment of herd immunity thresholds, while varying contact rates can measure the effectiveness of social distancing in reducing transmission. These models can provide critical insights into healthcare resource needs during epidemics. By estimating case numbers, they help forecast demands for medical staff, beds, and supplies during outbreak peaks (14). They also aid in understanding long-term disease prevalence trends influenced by immunity loss, seasonality, and demographic change (10, 15), supporting sustainable public health strategies.

Beyond these applications, compartmental models have been used to explore interactions between ecology and pathogen evolution, which influence disease emergence, persistence, and spread. They are instrumental in studying host–pathogen adaptation cycles, where pathogens evolve in response to interventions such as vaccines and therapeutics (16–18). Such models also provide a framework to evaluate specific hypotheses about pathogen evolution and persistence (19). Examples include the Red Queen Hypothesis, which frames host–pathogen interactions as an evolutionary “arms race” (20); phylodynamic approaches linking genetic change during transmission to epidemiological patterns (21); and antigenic drift and shift, which describe gradual and major changes in viral surface proteins, respectively (22). Modeling these processes is essential for understanding the rapid adaptation of pathogens such as influenza and their implications for public health (23).

Although few studies have used compartmental models for exploring various hypotheses associated with the evolution of different strains of pathogens (6, 16, 17), the utility of these models to test competing hypotheses has largely been overlooked. To fill this gap, the present study develops a framework to utilize compartmental models for testing eco-evolutionary hypotheses and decomposing the mutual influences of viral mutation and human interventions. As explained in the next section, we will use a model-based hypothesis testing approach to examine various adaptive strategies of pathogen, transmission, and immune evasion in the context of the COVID-19 pandemic.

### 1.2 Literature review on existing compartmental COVID-19 models

Compartmental models have been widely used to analyze COVID-19 transmission dynamics, the emergence of variants, and the impact of interventions. Several studies have addressed variant-specific dynamics. For example, Ngonghala and Taboe (24) modeled Delta and Omicron spread in the United States, showing that elimination requires high vaccination coverage along with widespread mask use, while de León et al. (25) analyzed multistrain interactions under immune waning and booster programs, identifying transmissibility and immune escape as key drivers of variant dominance. Other works examined the role of vaccination efficacy and cross-protection. Mancuso and Gumel (12) used a two-strain, two-group model to quantify herd immunity thresholds under varying transmissibility, and Gonzalez-Parra and Arenas (26) determined elimination thresholds for the wild-type strain in the absence of competing variants. Saha and Podder (27) combined vaccination with non-pharmaceutical interventions such as social distancing and quarantine, while other extensions incorporated co-morbidities and reinfection to evaluate control strategies in more realistic settings (28–30).

Recent developments have expanded these frameworks to capture additional biological and epidemiological complexity. Kim et al. (31) included multiple types of vaccination, mutant viruses, and breakthrough infections; Yang et al. (32) used an age-structured approach to jointly assess transmissibility and virulence; and Pant and Gumel (33) demonstrated that accounting for age heterogeneity lowers the vaccination threshold for herd immunity. Zelenkov and Reshettsov (34) applied genetic algorithms to reconstruct the transition rate distributions, allowing estimation of unobservable parameters such as vaccine protection and unregistered cases.

Methodological advances have also integrated machine learning to improve predictive accuracy. Baccega et al. (15) developed Sybil, a variant-aware compartmental modeling framework enhanced with machine learning, achieving more accurate short- and medium-term forecasts than traditional approaches. Other works have investigated intervention scenarios: Kosinski (35) modeled a hypothetical pan-coronavirus vaccine and highlighted the influence of behavioral factors on vaccine impact; Li et al. (13) evaluated how reduced vaccine effectiveness from variants affects safe social restoration; and Bates et al. (36) linked longer intervals between infection and vaccination to stronger cross-variant immunity.

Together, these studies (summarized in Table 2) have advanced our understanding of the dynamics of SARS-COV-2 disease and the effects of the interventions. However, despite these advances, to the best of our knowledge, no existing models have been used to rigorously test competing hypotheses related to different variants of SARS-CoV-2 and public health interventions. To bridge this gap, we followed the proposed framework illustrated in Figure 2. Our first step is to conduct a comprehensive literature review to systematically identify and formulate competing hypotheses, as outlined in the next section.

**Table 2:** Summary of key findings for SARS-CoV-2 evolution using compartmental models.

**References**	**Main result**
Mancuso and Gumel (12)	Herd immunity thresholds rise with variant transmissibility, requiring 59% vaccination for the wild-type strain, 76% for Alpha, and 82% for Delta.
Gonzalez-Parra and Arenas (26)	The variant with the higher reproduction number will dominate, regardless of lifelong immunity or vaccination programs, and the introduction of more transmissible variants accelerates their prevalence.
de León et al. (25)	Variant dominance depends on transmissibility and immune escape. The model accounts for multi-strain dynamics, immunity waning, and booster doses in managing pandemic waves.
Li et al. (13)	Achieving 70% vaccination coverage with 88.5% effectiveness can control COVID-19 and enable safe social restoration, but reduced effectiveness from variants like Delta may lead to new waves.
Kim et al. (31)	Early vaccination flattens the infection curve, delays peak cases, and reduces strain on healthcare systems, while higher vaccination rates significantly lower infections.
Ngonghala and Taboe (24)	Omicron emerges as the predominant variant, with Delta eventually dying out, and pandemic elimination requires a combination of increased vaccination coverage and widespread use of effective masks.
Bates et al. (36)	Longer infection-vaccination intervals (up to 400 days) enhance antibody titers and cross-variant protection. There is a possibility for hybrid immunity and improving long-term COVID-19 defense against emerging variants.
Zelenkov et al. (34)	Vaccination reduces infection risk by 65–70%, though effectiveness declines with new variants like Delta. There are regional differences in immunity duration and infection rates, with unregistered cases far exceeding registered ones.
Baccega et al. (15)	A multistrain model with a machine learning approach outperforms traditional models in forecasting COVID-19 trends, accurately predicting infection peaks, declines, and variant-driven outbreaks across multiple countries and scenarios.
Kosinski (35)	While the vaccine effectively reduces morbidity, its efficacy is significantly reduced by high-transmission variants, vaccine hesitancy, and the discontinuation of NPIs.
Pant and Gumel (33)	Using data from three pandemic waves dominated by Alpha, Delta, and Omicron variants, the study shows that strategies targeting asymptomatic and pre-symptomatic individuals are consistently effective.

### 1.3 Model-based hypothesis testing

Model-Based Hypothesis Testing (MBHT) is a framework that enables researchers to evaluate competing mechanistic hypotheses by integrating compartmental models with relevant data. In many settings, there is insufficient direct evidence to distinguish among hypotheses at the level of individual parameters or mechanisms. Unlike traditional hypothesis testing, which typically relies on a test statistic derived from observed data, MBHT leverages the output of a validated dynamical model that captures the underlying ecological, evolutionary, or epidemiological processes (37, 38). For example, even when direct measurements of disease transmission rates or virulence are unavailable, their distributions can be inferred by fitting a compartmental model to morbidity and mortality data. These inferred parameter distributions can then be used to quantify the relative support for alternative hypotheses. In this way, MBHT enhances the specificity and interpretability of inference by comparing hypotheses through model-based simulations rather than relying solely on empirical contrasts.

The MBHT approach differs fundamentally from classical hypothesis testing by offering a mechanistic, model-driven framework tailored to nonlinear, time-dependent systems. Conventional statistical methods typically compare outcomes between control and treatment groups, assuming that the relevant mechanisms can be probed directly from data. In contrast, MBHT relies on model analysis and simulation to identify the best-supported hypothesis, especially when key mechanisms are only indirectly observed (39). Algorithms such as Approximate Bayesian Computation, Markov Chain Monte Carlo, and machine-learning-based emulators can be used to align model output with observed data and to assess the robustness of conclusions. This makes MBHT particularly well suited for dynamic systems such as pathogen evolution, where feedbacks between transmission, immunity, and intervention strategies create complex adaptive and co-evolutionary dynamics that may not be adequately captured by standard statistical tests.

**Figure 1:**
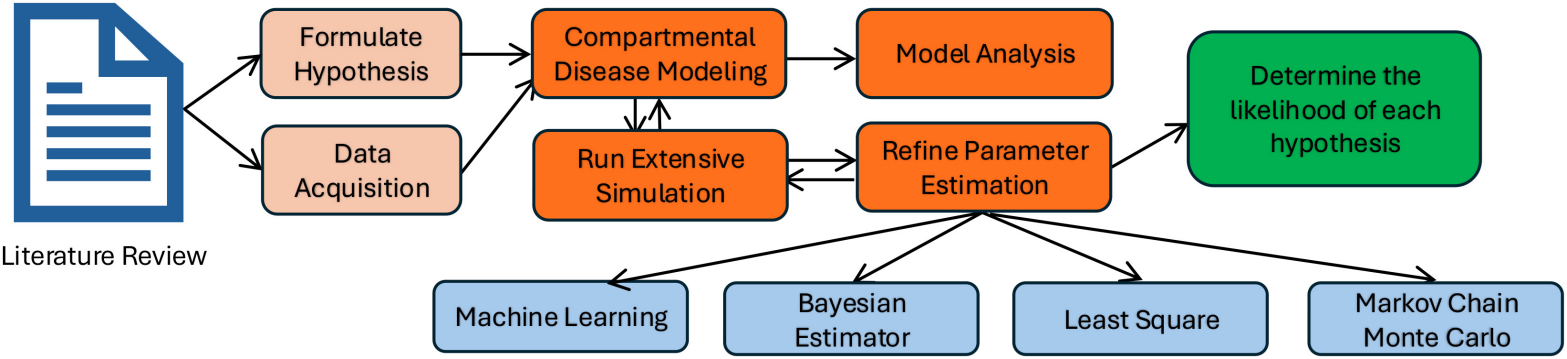
Flowchart of the Model-Based Hypothesis Testing (MBHT) approach. Using existing literature, hypotheses are formulated, and relevant data can be obtained (shown with a dashed arrow). A compartmental disease model is developed with defined parameter ranges. Through extensive sampling of parameter values and optimization techniques, such as least squares or Markov Chain Monte Carlo, parameter estimates are refined. The resulting parameter distributions are then used to evaluate the likelihood of competing hypotheses.

Figure 1 summarizes the typical workflow for applying MBHT to ecological and evolutionary systems or other complex dynamical settings. First, competing hypotheses are formulated on the basis of a comprehensive literature review and supplemented with data from relevant repositories. To test these hypotheses, a compartmental disease model is constructed, analyzed using standard mathematical tools, and simulated by sampling parameter values from specified prior ranges. The analysis yields key quantities, such as the basic reproduction number and bifurcation thresholds, which in turn guide the refinement of parameter estimates (e.g., transmission and virulence rates) through optimization or Bayesian calibration methods. The refined parameter distributions are then used to evaluate the likelihood of each competing hypothesis using statistical techniques such as Bayesian inference, likelihood ratio tests, or posterior probability estimation.

Beyond ecology, MBHT has been applied in a number of domains. In software engineering, Camilli et al.(40) used MBHT to assess the reliability of software systems under uncertain operating conditions, distinguishing among failure and recovery models and thereby improving system robustness. In systems biology, Cedersund et al. (41) applied MBHT to insulin signaling pathways in human adipocytes to identify plausible compartmental models that inform metabolic research. In studies of pathogen evolution, MBHT has contributed to modeling immune escape mechanisms in HIV, helping to identify promising vaccine targets (42). Ecologists have employed MBHT to study predator–prey dynamics and how environmental change alters species traits and community structure (43). In microbiology, MBHT has been used to analyze the evolution of antibiotic resistance, for example, by developing molecular ecological networks to characterize microbial interactions and assess how these interactions respond to warming or nutrient enrichment (44). Such applications illustrate how MBHT can reveal the relationships that sustain ecological networks and support biodiversity conservation under environmental change (44).

Despite its growing use in several fields, MBHT remains underutilized in studies of pathogen evolution that explicitly couple pathogen dynamics with public health interventions. In particular, there is still limited work that uses MBHT to quantify the relative contributions of evolutionary pressures such as immune evasion, transmission–virulence trade-offs, and intervention-based selection to long-term pathogen transmissibility and resilience to control measures (16, 45–48). In this study, we take mutations of SARS-CoV-2 as a case study and extend MBHT to evaluate competing eco–eco-evolutionary hypotheses across successive epidemic waves in the United States. In particular, the novelty in our proposed MBHT methodology lies in its integration of mechanistic disease modeling, statistical inference, and a machine-learning–style resampling scheme to estimate hypothesis likelihoods across successive epidemic waves. In particular, we use posterior probability estimation to quantify which eco–evolutionary hypothesis best explains each observed variant transition, providing a quantitative comparison across epidemic phases. Although we develop and illustrate this framework using COVID-19 in the United States, the same strategy can be applied more broadly to other pathogens and intervention scenarios. Reflecting the structure detailed in Figure 1, the remainder of this paper systematically follows the MBHT framework in the context of SARS-CoV-2 evolution. Section 1.2 provides a literature review of existing COVID-19 compartmental models, which serves as the basis for Section 2.1, where we formulate six specific hypotheses about how transmission, virulence, immunity, and vaccination pressures may have driven the evolution of COVID-19 variants in the United States. Section 2.2 then introduces the vaccination-structured SVEI_*a*_I_*s*_R compartmental model and examines its qualitative properties, including well-posedness, equilibrium states, the basic reproduction number *R*_0_, and the conditions under which backward bifurcation can occur. Section 3 implements the data collection–model fitting–parameter refinement part of Figure 1. Namely, we fit the model to U.S. case data for seven distinct epidemic waves, conduct local and global sensitivity analyses, and study how the estimated parameters and *R*_0_ values change from one wave to the next. Section 3.5 employs the MBHT framework to combine these parameter estimates and their uncertainties, computing posterior probabilities for each of the six hypotheses across wave-to-wave transitions, and Section 4 discusses how these results inform our understanding of SARS-CoV-2 evolution and the role of public health interventions.

## 2 Methods and materials

### 2.1 COVID-19 incidence and social distancing data

We used two types of data in this study. Confirmed COVID-19 case counts for the 314 United States counties obtained from the New York Times COVID-19 data repository,^1^ which were used to estimate parameter values of the model for each epidemic wave and social-distancing survey data from (49), which were used to estimate contact rates and probabilities of transmission. Details of these data and the related computations are provided in Sections 3.1, 3.4.

### 2.2 Formulating SARS-CoV-2 hypotheses

The rapid mutations of SARS-CoV-2 throughout the pandemic gave rise to multiple variants, each characterized by distinct mechanisms of transmission and immune evasion.

Notable variants such as Alpha, Delta, and Omicron have introduced mutations in the virus's spike protein, significantly enhancing its ability to infect hosts and, in some cases, evade vaccine-induced immunity (50, 51). For example, the Delta variant was characterized by increased transmissibility and high virulence, which contributed to severe waves of infection worldwide (24). In contrast, Omicron demonstrated a different behavior. While it was less virulent than Delta, it was highly transmissible and showed a strong capacity to evade neutralizing antibodies, even in vaccinated individuals (35, 51, 52). The mutations and their effects on transmission and virulence make SARS-CoV-2 an ideal example for studying pathogen evolution in real-time. The combination of high transmissibility, the emergence of multiple variants, and the selective pressure from global vaccination efforts provides a robust framework for analyzing viral evolution under varying public health conditions.

We identified six competing hypotheses associated with SARS-CoV-2, each offering different perspectives on the virus's evolutionary trajectory. Table 3 summarizes the description of those six hypotheses. In the next section, we present the compartmental model developed for testing the likelihood of the competing hypotheses.

**Table 3:** Overview of six competing hypotheses on pathogen mutation in SARS-CoV-2 evolution along with the references that include theoretical concepts.

***H*_*i*_**	**Hypothesis**	**Description**	**References**
H1	Transmission–virulence trade-off	Classical trade-off theory predicts evolution toward an intermediate virulence level, where transmission is maximized without killing hosts too quickly. Dynamically, this implies that increases in transmissibility are accompanied by stable or reduced virulence (or vice versa).	(18, 47)
H2	Short-term within host selection	The natural selection on viral replication within individual hosts favors variants that grow rapidly and reach high viral loads, thereby increasing both transmissibility and disease severity in the short term. As a result, we expect transient dominance of highly transmissible, highly virulent variants that are later replaced by other lineages.	(17, 79, 80)
H3	Vaccination-induced virulence	Vaccination that reduces disease severity more than transmission can create population-level selective pressure favoring variants with higher virulence (δ). Vaccinated hosts may act as partial reservoirs, allowing such variants to spread despite reduced symptomatic disease.	(52, 81)
H4	Immune selection	As infection- and vaccine-induced immunity accumulate, variants that escape existing antibody responses are favored. This leads to sequential replacement by immune-evasive strains, typically through mutations in key antigenic regions such as the spike protein.	(50, 51)
H5	Declining virulence	The declining virulence posits that, over a longer time period, successful pathogens evolve toward lower virulence while retaining sufficient transmissibility to persist. This predicts a gradual shift to variants that cause milder disease, particularly in populations with increasing immunity.	(47, 82)
H6	Transmission–virulence correlation	This hypothesis assumes that transmissibility and virulence tend to move in the same direction, rather than being constrained by a trade-off. We therefore expect variants with higher transmission also to show higher severity (or both reduced).	(18, 83)

### 2.3 The proposed model

Although spatial heterogeneity can affect SARS-CoV-2 transmission and evolution, we assume homogeneous spread of variants in our modeling approach because reliable, variant-specific *case counts* at regional or population-stratified levels are not available. U.S. CDC genomic surveillance reports variant information primarily as *proportions* of sequenced specimens (estimated nationally, by HHS region, and by jurisdiction), rather than complete case totals (53–55). Because sequencing covers only a small, uneven subset of infections across jurisdictions, these proportional estimates cannot be robustly converted into absolute numbers suitable for parameterizing a spatially or demographically stratified model. We therefore analyze aggregated population-level dynamics and emphasize temporal changes in transmission and virulence rather than geographic or demographic stratification.

**Figure 2:**
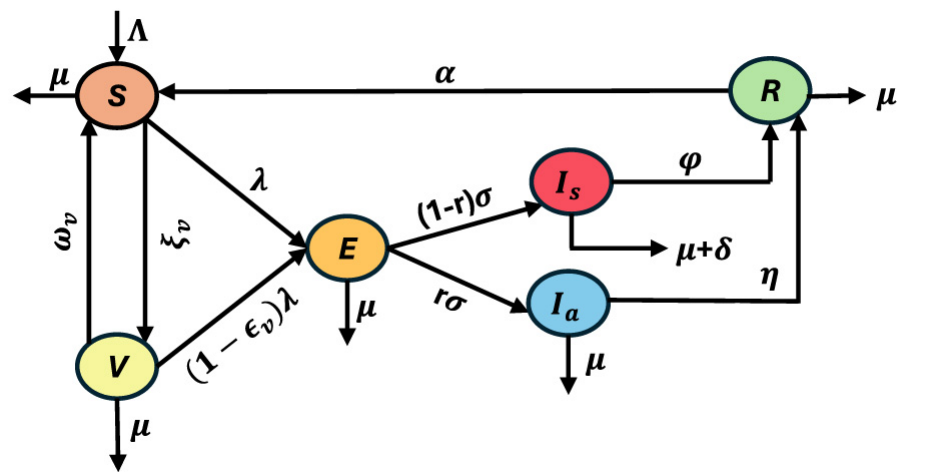
Schematic diagram of the proposed COVID-19 transmission model detailing the dynamics of COVID-19 transmission and control measures with the compartments Susceptible (*S*), Vaccinated (*V*), Exposed (*E*), Asymptomatic Infected (*I*_*a*_), Symptomatic Infected (*I*_*s*_), and Recovered (*R*). Key processes are depicted with arrows: recruitment into the susceptible population (Λ), vaccination (ξ_*v*_), waning of vaccine-induced immunity (ω_*v*_), infection of susceptible (β_*s*_, β_*a*_) and vaccinated individuals (1−ϵ_*v*_), progression from exposed to asymptomatic or symptomatic classes (*rσ*, (1−*r*)σ), recovery rates (η, ϕ), natural mortality (μ), disease-induced mortality (δ), and loss of immunity (α).

Given the above explanations we divide the host population into six epidemiological compartments: susceptible *S*(*t*), vaccinated *V*(*t*), exposed *E*(*t*), asymptomatic infected *I*_*a*_(*t*), symptomatic infected *I*_*s*_(*t*), and recovered *R*(*t*) individuals at time *t*. The interactions among these compartments are illustrated in Figure 2 and Table 4 summarizes the model variables, parameters, and their respective units considered in this study.

**Table 4:** The upper and lower bounds for parameters of model (Equation 1) derived from the existing literature along with their description and units. Parameters related to vaccination (ξ_*v*_, ϵ_*v*_, and ω_*v*_) were set to zero for cases where vaccination was not applicable.

**Symbol**	**Description**	**Unit**	**(Min, Max)**	**Reference**
Λ	Recruitment rate of susceptible individuals	Individual/day	(0.0095, 0.0117)	(84)
β_*s*_	Transmission rate of infected symptomatic class	Day^−1^	(0.0031, 0.3577)	(33)
β_*a*_	Transmission rate of infected asymptomatic class	Day^−1^	(0.0021, 0.4650)	(33)
μ	Natural mortality rate in all classes	Day^−1^	(0.0003, 0.0004)	(84)
ξ_*v*_	Vaccination rate	Day^−1^	(0.0024, 0.0601)	(85)
ϵ_*v*_	Vaccine efficacy	−	(0.61182, 0.9415)	(85)
ω_*v*_	Rate of decrease in vaccine-induced immunity	Day^−1^	(0.0004, 0.0100)	(24)
*r*	Proportion of individuals becoming asymptomatically infectious	−	(0.02341, 0.6534)	(24)
σ	Rate of progression to infectious class	Day^−1^	(0.0632, 0.5208)	(24)
η	Recovery rate of infected asymptomatic class	Day^−1^	(0.0316, 0.2214)	(24)
ϕ	Recovery rate of infected symptomatic class	Day^−1^	(0.0218, 0.1561)	(24, 86)
α	Loss of immunity rate	Day^−1^	(0.0037, 0.0125)	(33)
δ	Disease-induced mortality rate for symptomatic class	Day^−1^	(0.00015, 0.0019)	(33)

In our model, new individuals enter the susceptible class at the recruitment rate Λ. Susceptible individuals may receive vaccination at rate ξ_*v*_, moving to the vaccinated class, where the vaccine provides partial protection with efficacy ϵ_*v*_ ∈ (0, 1). Immunity induced by vaccination wanes at the rate ω_*v*_, after which individuals return to the susceptible class. Both susceptible and vaccinated individuals may become infected upon contact with infectious individuals, with the force of infection given by $\lambda =\frac{\beta_{s}I_{s}+\beta_{a}I_{a}}{N}$, where β_*s*_ and β_*a*_ are the effective contact rates of symptomatic and asymptomatic infected individuals, respectively. Newly infected individuals enter the exposed class *E*(*t*), where they remain for the incubation period and then progress to the infectious stage at the average rate σ. A fraction *r* of exposed individuals become asymptomatic infected *I*_*a*_(*t*), while the remaining fraction 1−*r* develop symptoms and move to the symptomatic class *I*_*s*_(*t*). Asymptomatic individuals are able to transmit infection but experience negligible mortality from the disease; they recover at rate η. Symptomatic individuals, on the other hand, may recover at rate ϕ or die due to the disease at rate δ. Individuals who recover, whether from the asymptomatic or symptomatic class, enter the recovered compartment *R*(*t*). However, immunity is not permanent based on the COVID-19 scenario, and reinfection has been observed in recovered individuals who can enter the susceptible compartment again at the rate α. In addition, individuals in each compartment experience natural mortality at rate μ.

The following system of non-linear ordinary differential equations provides the model equations:


$$\begin{array}{l} \frac{dS}{dt}=\text{Λ}+\omega_{v}V+\alpha R-\frac{\beta_{s}I_{s}+\beta_{a}I_{a}}{N}S-\left(\mu +\xi_{v}\right)S\\ \frac{dV}{dt}=\xi_{v}S-\left(1-\epsilon_{v}\right)\frac{\beta_{s}I_{s}+\beta_{a}I_{a}}{N}V-\left(\mu +\omega_{v}\right)V\\ \frac{dE}{dt}=\frac{\beta_{s}I_{s}+\beta_{a}I_{a}}{N}S+\left(1-\epsilon_{v}\right)\frac{\beta_{s}I_{s}+\beta_{a}I_{a}}{N}V-\left(\mu +\sigma \right)E\\ \frac{dI_{a}}{dt}=r\sigma E-\left(\mu +\eta \right)I_{a}\\ \frac{dI_{s}}{dt}=\left(1-r\right)\sigma E-\left(\mu +\delta +\phi \right)Is\\ \frac{dR}{dt}=\eta I_{a}+\phi I_{s}-\left(\mu +\alpha \right)R, \end{array}$$ (1)

where all parameters are non-negative constants and the initial conditions of model (Equation 1) satisfy the following inequalities:


$$S(0)>0,V(0)\geq 0,E(0)>0,{\text{I}}_{\text{a}}(0)>0,{\text{I}}_{\text{s}}(0)>0,R(0)\geq 0.$$ (2)

To evaluate the six hypotheses outlined in the previous section, it is essential to first confirm that the proposed model is well-defined and establish the conditions governing its potential dynamics. The next section presents a detailed analysis of the model.

## 3 Results

### 3.1 Well-posedness, stability, and thresholds

First, we establish the mathematical well-posedness of the model by demonstrating that its solutions remain positive and bounded for all time with the initial condition defined in Equation 2, which can ensure the biological feasibility of the model (Equation 1). Then, we examine the stability of the equilibrium solutions and derive key threshold quantities, including the basic reproduction number (*R*_0_) and the conditions for backward bifurcation (56, 57). These thresholds provide essential indicators of disease dynamics and will be used in subsequent sections to assess the competing hypotheses. For model well-posedness let total host population at time *t* be *N*(*t*) = *S*(*t*)+*V*(*t*)+*E*(*t*)+*I*_*a*_(*t*)+*I*_*s*_(*t*)+*R*(*t*). Adding all equations of model (Equation 1) gives $\frac{dN}{dt}\leq \text{Λ}-\mu N,$ which implies that ${\mathrm{limsup}}_{t\to \infty }N\left(t\right)\leq \frac{\text{Λ}}{\mu }$. Hence, the feasible region is defined by


$$\begin{array}{l} \text{Ω}=\left\{\left(S,V,I_{a},I_{s},R\right)\in {\mathbb{R}}_{+}^{5}|S+V+I_{a}+I_{s}+R\leq \frac{\text{Λ}}{\mu }\right\}, \end{array}$$ (3)

which is bounded and positively invariant (i.e., any trajectory starting in the set remains in the set for all future time). For details of model well-posedness, please see Supplementary Theorems 1, 2 in Section 1.

Next, we establish conditions for the existence and local stability of equilibrium solutions of the model (Equation 1) and calculate the basic reproduction number (*R*_0_). Similar to most disease models, model (Equation 1) may have two types of equilibria: a disease-free equilibrium (DFE) and an endemic equilibrium (EE), which are obtained by setting the right side of Equation 1 equal to zero. We get that


$$\begin{array}{l} DFE=\left(\frac{\text{Λ}\left(\mu +\omega_{v}\right)}{\mu \left(\mu +\omega_{v}+\xi_{v}\right)},\frac{\xi_{v}\text{Λ}}{\mu \left(\mu +\omega_{v}+\xi_{v}\right)},0,0,0,0\right), \end{array}$$ (4)

where the first two components correspond to the number of susceptible and vaccinated individuals, respectively. To investigate the local stability of the DFE (Equation 5, we first derive the basic reproduction number, *R*_0_, using the next-generation matrix approach (see the details in Supplementary Section 2). The *R*_0_ expression can be formulated as


$$\begin{array}{l} \;R_{0}=\gamma rR_{0}^{a}+\gamma \left(1-r\right)R_{0}^{s}\\ \text{where, }R_{0}^{s}=\frac{\beta_{s}}{\mu +\delta +\phi },R_{0}^{a}=\frac{\beta_{a}}{\mu +\eta },\gamma =\frac{\sigma \left(\mu +\omega_{v}+\xi_{v}\left(1-\epsilon_{v}\right)\right)}{\left(\mu +\sigma \right)\left(\mu +\omega_{v}+\xi_{v}\right)}. \end{array}$$ (5)

The terms $R_{0}^{a}$ and $R_{0}^{s}$ correspond to the contribution of asymptomatic and symptomatic individuals, respectively. Therefore, *R*_0_ is expressed as the weighted sum of $R_{0}^{a}$ and $R_{0}^{a}$. The value of *r* is between zero and one, which balances the contribution of the asymptomatic and symptomatic sub-populations in the spread of disease. The scaling factor γ adjusts the total *R*_0_ value by accounting for progression from exposed to infected, vaccination rate, waning immunity, and natural mortality.

In our study, *R*_0_ is an important measure. By applying the Routh–Hurwitz criterion, we establish that the disease-free equilibrium of system (Equation 1) is locally asymptotically stable in the feasible region Ω when *R*_0_ < 1 and unstable when *R*_0_>1 (see Supplementary Theorem 3 in Section 3). We further use *R*_0_ in both local and global sensitivity analyses to identify parameters with the greatest influence on disease transmission. Within our hypothesis testing framework, comparing *R*_0_ values across seven COVID-19 waves enables us to track changes in transmissibility over time and to infer whether viral evolution, public health interventions, or both most likely drove these shifts.

After examining the stability of DFE, we explore conditions under which the infection persists in the population (i.e. when the endemic equilibrium (EE) exists and is stable). We obtain analytical expressions for the EE (full derivation provided in Supplementary Theorem 4, Supplementary Section 4). Mathematically, the expression for the EE can be reduced to a quadratic condition whose coefficients determine whether a unique, multiple, or no endemic equilibrium is possible under different conditions (see Supplementary Theorem 5). The case where two endemic equilibria exist even when *R*_0_ < 1 shows the possibility of a backward bifurcation.

In our model, we establish the condition for the existence of backward bifurcation. This condition shows that, when the vaccination rate ξ_*v*_ is less than a critical threshold $\xi_{v}^{*}$, which is defined as follows, the phenomenon of backward bifurcation occurs. (see the full proof in Supplementary Theorem 5 by using the center manifold theorem in Supplementary Section 5). The critical threshold is defined by


$$\begin{array}{l} \xi_{v}^{*}=\frac{1+f\left(\mu ,\omega_{v},w_{i}\right)}{\epsilon_{v}w_{1}+\left(1-\epsilon_{v}\right)(1+w_{2}f\left(\mu ,\omega_{v},w_{i}\right))} \end{array}$$ (6)

where *f*(μ, ω_*v*_, *w*_*i*_) = (μ+ω_*v*_)(*w*_4_+*w*_5_+*w*_6_). The coefficients *w*_*i*_ (*i* = 1, …, 6) are the components of the eigenvector corresponding to the zero eigenvalue of the Jacobian matrix evaluated at the endemic equilibrium. Higher values of μ or ω_*v*_ increase *f*, which in turn raises the threshold $\xi_{v}^{*}$ and makes backward bifurcation less likely for the same vaccination coverage. Two important special cases illustrate the implications of this threshold: (i) If *w*_2_ = 1 and ϵ_*v*_≈0 (a nearly ineffective vaccine), then $\xi_{v}^{*}\approx 1$, which becomes a baseline value independent of model parameters; (ii) if *f* → ∞ (i.e., rapid waning in vaccination immunity or extremely high natural mortality ), then


$$\begin{array}{l} \xi_{v}^{*}\approx {\left[w_{2}\left(1-\epsilon_{v}\right)\right]}^{-1}=\frac{R_{u}}{w_{2}R_{v}} \end{array}$$ (7)

where *R*_*u*_ and *R*_*v*_ represent the infection risks in unvaccinated and vaccinated sub-populations, respectively. In any of these cases, the inequality $\xi_{v}^{*}>\xi_{v}$, highlights the importance of achieving sufficiently high vaccination rates to avoid the scenario of backward bifurcation, where low vaccination coverage could allow the infection to become endemic. These findings are consistent with the challenges encountered in achieving herd immunity during the COVID-19 pandemic.To numerically explore the bifurcation threshold, we focus on the effects of the symptomatic transmission rate. As shown in Supplementary Figure S1, the backward bifurcation dynamics in the vaccinated (*V*) and symptomatic infected (*I*_*s*_) sub-populations change according to the values of the symptomatic transmission rate. As β_*s*_ increases, the system moves from a stable disease-free state to a bistable region where both disease-free and endemic equilibria can occur. This highlights the importance of maintaining vaccination rates above the threshold $\xi_{v}^{*}$ to prevent persistence of infection even when *R*_0_ < 1.

To further support these findings, we rigorously establish the global stability of both the disease-free and endemic equilibria under the assumptions of no reinfection and fully effective vaccination. When the efficacy of the vaccine is perfect (ϵ_*v*_ = 1) and *R*_0_ < 1, the disease-free equilibrium (DFE) is globally asymptotically stable, implying the elimination of infection regardless of the initial conditions. Conversely, when *R*_0_>1 under the same assumptions, the endemic equilibrium (EE) is globally asymptotically stable. Complete mathematical proofs of these results are provided in Supplementary Section 6 in Theorem 6. After establishing all of the mathematical properties of the model, we are now positioned to perform the hypothesis testing using the validated model.

### 3.2 Model parametrization

**Figure 3:**
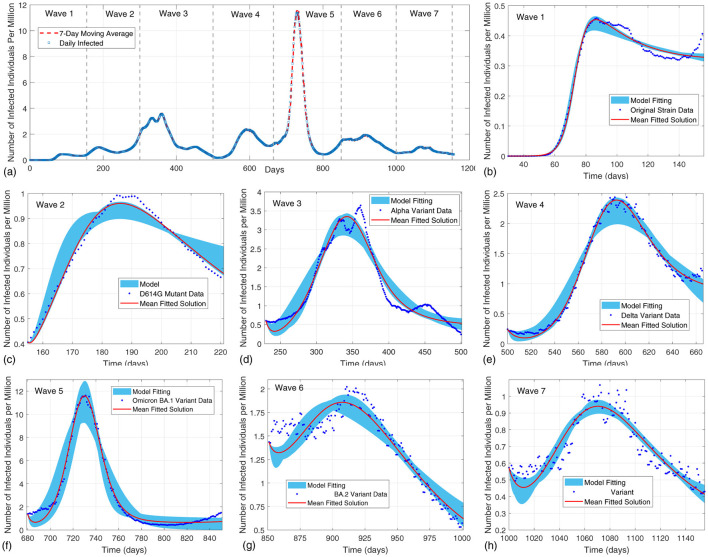
Model solutions fitted to COVID-19 data across seven waves in the US. **(a)** Daily infection data with 7 days moving average per million. **(b–h)** Model fits for each wave (specific variants) with observed data (blue), mean predictions (red), and variability region (shaded) in model predictions due to differences in parameter estimates across simulations which collectively highlight variant-specific trends.

Building on the theoretical properties of the model, this subsection applies the model-based hypothesis testing (MBHT) approach to analyze the evolutionary dynamics of SARS-CoV-2 across seven epidemic waves, serving as a case study. We obtained daily confirmed COVID-19 case counts for the United States from the New York Times COVID-19 data repository (see footnote 1). The incidence curve was smoothed using a 7-day moving average, and then seven epidemic waves were defined by visually identifying distinct rising and falling phases in this smoothed time series. The resulting wave boundaries were cross-checked against CDC reports on periods of dominance for major SARS-CoV-2 variants to ensure consistency (see Figure 3a). We defined the ranges of the model (Equation 1) parameter values based on the existing literature. These ranges, detailed in Table 4, were used as constraints during the model fitting process. For the vaccination parameters, we updated their values from zero to positive once vaccines became available. Specifically, the vaccination rate ξ_*v*_, vaccine efficacy ϵ_*v*_, and waning rate ω_*v*_ were fixed at zero for Waves 1 and 2 to reflect the absence of vaccination in the early pandemic phase, and were allowed them to vary within the bounds defined in Table 4 for subsequent waves. For all other parameters, we did not impose any a priori pattern of change. Instead, we fitted the model separately to each epidemic wave using the same literature-informed parameter ranges summarized in Table 4. As a result, parameter estimates are allowed to differ between waves, and any abrupt changes at wave boundaries arise from the data-driven simulation. Moreover, using additional data extracted from Gallup (49), we further decomposed the fitted transmission rates β_*s*_ and β_*a*_ into contact rates *C*_*s*_ and *C*_*a*_ and infection probabilities *p*_*s*_ and *p*_*a*_ to separate behavioral and biological drivers of transmission (see Section 3.4). We use these decomposed parameters in the calculation of the posterior probabilities for the competing hypotheses.

To fit the model to observed infection data, we utilized MATLAB fmincon from the optimization toolbox to minimize the sum of squared residuals (SSE) between the model-predicted and observed values. The goodness of fit was evaluated using the coefficient of determination (*R*^2^). These values are more than 0.7 for every simulation across the seven waves. This validation step ensured that the model was sufficiently flexible to capture the diverse dynamics of different variants while maintaining consistency with the observed data (Supplementary Figure S4).

Figure 3a presents the daily number of infected individuals per million across seven distinct epidemic waves. Specifically, the COVID-19 epidemic waves were defined as follows: Wave 1 (February 18, 2020–June 22, 2020), Wave 2 (June 22, 2020–September 5, 2020), Wave 3 (September 5, 2020–June 2, 2021), Wave 4 (June 2, 2021–November 30, 2021), Wave 5 (November 30, 2021–May 18, 2022), Wave 6 (May 18, 2022–October 16, 2022), and Wave 7 (October 16, 2022–March 22, 2023). The variability in peak magnitudes, durations, and infection declines reflects the evolving interplay between viral adaptations, population immunity, and public health measures. Figures 3b–h shows the observed data (dotted blue curves), mean fitted model solutions (solid red curves), and shaded regions representing the variability in the model (Equation 1) predictions due to variations in parameter estimates. It can be seen that there is a close alignment between the model predictions and the observed data. The shaded bands in Figures 3b–h represent the variability region of the model solutions arising from uncertainty in the parameter values. For each wave, we repeatedly solved model (Equation 1) for 70,000 parameter combinations (10,000 per wave) sampled within the ranges given in Table 4. This ensemble of simulations produces a family of model solutions, and the shaded region shows the variance of these solutions at each time point. Consequently, the width of the shaded band reflects how sensitive the predicted number of infections is to plausible changes in the transmission, recovery, and immunity parameters: narrow bands indicate that the data constrain the parameters tightly, whereas wider bands indicate that several different parameter combinations can reproduce the observed epidemic curve. In Wave 1 (Figure 3b), the original strain shows a gradual increase and decrease in infections, with the shaded region demonstrating a narrow range of uncertainty, reflecting the consistency of early pandemic data. The transition to Wave 2 (Figure 3c), driven by the D614G mutation, reveals a sharper infection peak and broader uncertainty bounds, highlighting the variant's enhanced transmissibility and the challenges in modeling the rapid changes. Wave 3 (Figure 3d), dominated by the Alpha variant, features a significantly higher peak compared to Wave 2 and a wider shaded region, indicating more significant uncertainty associated with the variant's immune evasion and transmission dynamics. The shift to Wave 4 (Figure 3e), driven by the Delta variant, demonstrates a steep rise and fall in infections, with the shaded region narrowing, suggesting reduced variability in the data due to stronger control measures and vaccination efforts. The progression to Wave 5 (Figure 3f), associated with the Omicron BA.1 variant, shows the highest infection peak among all waves, signifying the variant's immune escape capabilities and the rapid spread within a partially vaccinated population. After transitioning to wave 6 (Figure 3g), dominated by Omicron BA.2, it exhibits a moderate peak and a prolonged duration, highlighting the sustained transmission potential despite accumulated immunity. The final wave 7 (Figure 3h), according to our datasets, characterized by the variant XBB1.6, shows a moderate peak with a slower decline, reflecting advanced immune evasion and the interaction between viral evolution and population-level immunity.

### 3.3 Local and global sensitivity analyses

In this subsection, We conducted a local and global sensitivity analysis to assess the relative influence of each parameter on predicting extreme values of *R*_0_ within individual epidemic waves. By applying Local Sensitivity Analysis (LSA) (58), we verified that β_*a*_ and β_*s*_ linearly increase $R_{0}$ values. Whereas η, ϕ, and δ appear in the denominators of the transmission terms and thus hyperbolically decrease $R_{0}$ values.

The elasticity index [a.k.a. normalized sensitivity index (58)] measures the relative change of *R*_0_ with respect to a parameter ω, denoted by ${\text{Γ}}_{R_{0}}^{\omega }$, and is defined as


$$\begin{array}{l} {\text{Γ}}_{R_{0}}^{\omega }=\frac{\partial R_{0}}{\partial \omega }\frac{\omega }{R_{0}}. \end{array}$$ (8)

The sign of ${\text{Γ}}_{R_{0}}^{\omega }$ indicates whether *R*_0_ increases (positive) or decreases (negative) with the parameter, while its magnitude determines the parameter's relative importance in driving changes in $R_{0}$. Using the elasticity index, we establish analytical conditions under which one parameter is more influential than another in increasing or decreasing $R_{0}$. In particular, the transmission rate of asymptomatic individuals is more influential than that of symptomatic individuals (i.e., ${\text{Γ}}_{R_{0}}^{\beta_{a}}>{\text{Γ}}_{R_{0}}^{\beta_{s}}$) if


$$\begin{array}{l} \frac{\beta_{a}}{\beta_{s}}>\left(\frac{1-r}{r}\right)\frac{\mu +\eta }{\mu +\delta +\phi }, \end{array}$$ (9)

where 0 < *r* ≤ 1. Conversely, the transmission rate of symptomatic individuals is more influential when the inequality is reversed. This can happen when the values of *r* are sufficiently small (i.e., close to 0).

Similarly, the recovery rate of asymptomatic individuals is more influential than that of symptomatic individuals (i.e., ${\text{Γ}}_{R_{0}}^{\eta }>{\text{Γ}}_{R_{0}}^{\phi }$) if


$$\begin{array}{l} \frac{\eta }{\phi }>\left(\frac{1-r}{r}\right)\frac{\beta_{s}}{\beta_{a}}{\left(\frac{\mu +\eta }{\mu +\delta +\phi }\right)}^{2}. \end{array}$$ (10)

The influence of ϕ is greater than η if the above inequality is reversed. The detailed proofs of these results are provided in Supplementary Theorems 8, 9 of Section 8. The same analytical approach can be applied to any other set of parameters.

While LSA is a powerful tool for identifying the influence of individual parameters and enabling direct pairwise comparisons, it is inherently limited to behavior around nominal parameter values. As such, these results may not generalize across the full parameter space. Therefore, we carry out a Global Sensitivity Analysis (GSA), as outlined below.

We applied GSA by focusing on *R*_0_ anomalies within each epidemic wave. The *R*_0_ values used in the GSA were computed from our model using its analytic expression for *R*_0_ that has been derived in the equation (Equation 6). For each wave, we generated 10,000 parameter sets using a Monte Carlo simulation framework, where each model parameter was drawn independently from a uniform distribution bounded by its predefined lower and upper limits based on literature values. These simulations allowed us to compute 10,000 corresponding *R*_0_ values per wave. To identify anomalies, we calculated the Z-score of each simulated *R*_0_ value and labeled those with Z-scores greater than 1.96 as anomalies. This binary anomaly label (1 for extreme, 0 for normal) was used as the target variable in a Classification and Regression Tree (CRT) analysis performed in SPSS. The model parameters were used as input features, and the CRT algorithm provided normalized importance scores indicating the relative influence of each parameter in generating extreme *R*_0_ values. The CRT demonstrated robust predictive capability, with overall accuracy exceeding 95% in most intervals. Sensitivity and specificity remained consistently high (see Supplementary Table S1).

Table 5 identifies the mechanisms driving SARS-CoV-2 transmission across each wave by summarizing the effects of parameter change on $R_{0}$. The first two rows correspond to local sensitivity analysis and show the proportion of simulations in which the conditions (Equations 11, 12) are satisfied. In particular, large values in these rows indicate that asymptomatic transmission (β_*a*_) and recovery of asymptomatic individuals (η) exert greater control over $R_{0}$ than the corresponding symptomatic parameters. In Waves 3, 5 these proportions are particularly high, which coincides with the fact that during the emergence of Alpha (Wave 3) and especially Omicron BA.1 (Wave 5), epidemic growth was strongly influenced by infections with few or no symptoms [i.e., through silent onward transmission and rapid turnover in the asymptomatic or mildly symptomatic class (59)]. In contrast, the much lower percentages in Waves 1, 2, 4, and 6 indicate that symptomatic transmission and symptomatic recovery dominate the behavior of $R_{0}$ in those periods.

**Table 5:** Local and Global sensitivity analyses of model parameters across seven COVID-19 waves using the CRT method. The first two rows are associated with the local sensitivity analysis and show the percentage of the cases that conditions in Equations 11, 12 are satisfied, respectively. The remaining rows are associated with the global sensitivity analysis and show normalized importance values for each parameter and the associated with percentage importance values shown in parentheses. The boldface entries indicate the most influential parameters per wave. For waves 1 and 2, ω, ϵ_*v*_, and ξ_*v*_ were set to zero due to absence of vaccination.

**Parameter**	**Wave 1**	**Wave 2**	**Wave 3**	**Wave 4**	**Wave 5**	**Wave 6**	**Wave 7**
${\text{Γ}}_{R_{0}}^{\beta_{a}}>{\text{Γ}}_{R_{0}}^{\beta_{s}}$	21.4 %	39.2%	**94.6%**	49.4%	**53.3%**	44.8%	13.1%
${\text{Γ}}_{R_{0}}^{\eta }>{\text{Γ}}_{R_{0}}^{\phi }$	21.17%	49.4%	**98.4%**	72.4%	**90%**	48.1%	16.2%
β_*a*_	**55.0% (0.034)**	**68.6% (0.095)**	**57.8% (0.066)**	9.0% (0.011)	2.9% (0.005)	17.2% (0.025)	19.4% (0.016)
δ	36.3% (0.022)	**66.6% (0.092)**	37.5% (0.043)	20.0% (0.025)	7.3% (0.012)	8.5% (0.013)	**88.2% (0.074)**
*r*	**52.9% (0.032)**	**71.4% (0.099)**	19.5% (0.022)	26.4% (0.033)	7.3% (0.012)	**93.5% (0.138)**	**91.4% (0.084)**
β_*s*_	**100.0% (0.061)**	**99.8% (0.138)**	22.8% (0.026)	**100.0% (0.125)**	25.7% (0.042)	**100.0% (0.148)**	**71.4% (0.060)**
μ	40.0% (0.024)	47.9% (0.066)	9.5% (0.011)	10.0% (0.013)	7.8% (0.013)	49.6% (0.073)	36.3% (0.031)
Λ	35.6% (0.022)	42.6% (0.059)	10.5% (0.012)	6.5% (0.008)	4.5% (0.007)	**61.2% (0.090)**	**66.5% (0.056)**
ϕ	37.8% (0.023)	**88.5% (0.122)**	**54.3% (0.062)**	19.1% (0.024)	3.8% (0.006)	4.6% (0.007)	**68.9% (0.025)**
σ	**75.6% (0.046)**	**100.0% (0.138)**	11.0% (0.013)	42.5% (0.053)	25.0% (0.041)	3.2% (0.005)	12.4% (0.010)
α	40.3% (0.025)	65.6% (0.091)	53.1% (0.061)	8.2% (0.010)	6.0% (0.010)	51.3% (0.076)	45.3% (0.038)
η	48.8% (0.030)	**61.8% (0.085)**	**95.8% (0.114)**	8.9% (0.011)	43.9% (0.071)	**63.7% (0.094)**	40.0% (0.034)
ξ_*v*_	−−	−−	13.3% (0.015)	45.5% (0.057)	6.8% (0.011)	**61.1% (0.090)**	39.7% (0.033)
ϵ_*v*_	−−	−−	26.5% (0.030)	20.5% (0.026)	**95.9% (0.155)**	41.3% (0.061)	33.6% (0.028)
ω	−−	−−	24.9% (0.029)	23.2% (0.029)	**100.0% (0.162)**	**51.2% (0.076)**	42.7% (0.036)

The remaining rows present normalized importance scores from the global sensitivity analysis and show which parameters are most influential within each wave. In this Table 5, we work directly with the fitted effective transmission rates β_*s*_ and β_*a*_, because the goal is to quantify how these composite rates contribute to variations in *R*_0_ across waves.Across all waves, the transmission rates (β_*s*_, β_*a*_), the proportion of individuals progressing to asymptomatic infection (*r*), and the recovery rate of symptomatic individuals (ϕ) are repeatedly identified as key drivers of infection and main sources of variability in $R_{0}$. In waves 1 and 2, the elevated importance of β_*s*_ indicates that symptomatic transmission played a more significant role because asymptomatic infections were less common and less infectious than in later waves, which aligns with the existing literature (60). Also, the importance of β_*s*_ extends to waves 4, 6. In the waves (5–7), the increasing importance of mortality and immunity-related parameters (δ, α, ω, ϵ_*v*_, and ξ_*v*_) shows that disease severity, waning immunity, and vaccination increasingly influence the disease dynamics and $R_{0}$ values.

### 3.4 Analysis of estimated values

After fitting model (Equation 1) to time series data of each wave, we now examine how the estimated epidemiological parameters change across the COVID-19 waves. To evaluate this, we calculated Cliff's Delta (CD) and the percentage change in mean parameter values between consecutive waves. Table 6 summarizes the observed variations in transmission, immunity loss, mortality, and vaccination-related factors. A Cliff's Delta (CD) value of 1 indicates that all values in wave *W*_*i*_ exceed those in *W*_*i*+1_, and a value of –1 indicates the reverse (61). According to standard thresholds, |*CD*| < 0.147 is negligible, 0.147 ≤ |*CD*| < 0.33 is small, 0.33 ≤ |*CD*| < 0.474 is medium, and |*CD*|≥0.474 is large.

**Table 6:** Mean percentage change and Cliff's Delta (CD) values for model parameters across consecutive COVID-19 epidemic waves. Transitions between waves are denoted as *W*_1_ to *W*_2_, *W*_2_ to *W*_3_, and so forth. Bolded values highlight the most significant parameter changes observed during each wave transition.

**P**.	***W*_1_−*W*_2_**	***W*_2_−*W*_3_**	***W*_3_−*W*_4_**	***W*_4_−*W*_5_**	***W*_5_−*W*_6_**	***W*_6_−*W*_7_**
*R*_0_	**–59.7% (1.0)**	–13.9% (0.1)	–44.7% (0.8)	**94.0% (–0.7)**	**–65.6% (0.8)**	27.9% (-0.6)
δ	**342.8% (–0.8)**	**86.5% (-0.9)**	–42.5% (0.8)	**308.0% (–1.0)**	**–97.0% (1.0)**	**146.1% (–0.8)**
*p*_*s*_	**75.4%** (–0.8)	**–76.8%** (0.8)	5.9% (-0.1)	**98.8%** (–0.7)	–79.2% (0.9)	41.8% (–0.4)
*p*_*a*_	10.1% (1.0)	–31.8% (0.9)	–21.2% (0.3)	18.4% (0.0)	–27.5% (0.3)	–18.9% (0.2)
ω_*v*_	None (–)	NA (0.2)	31.5% (–0.2)	38.1% (–0.7)	–63.7% (0.7)	**84.0%** (–0.7)
α	–70.2% (1.0)	62.9% (–1.0)	71.9% (–1.0)	–40.4% (0.5)	–12.7% (–0.3)	50.4% (–0.9)
σ	31.2% (–1.0)	**–90.4%** (1.0)	15.7% (–0.2)	55.1% (–0.7)	0.1% (0.1)	–22.5% (0.2)
*r*	26.2% (–1.0)	–46.6% (1.0)	–58.0% (1.0)	59.1% (–0.5)	–18.0% (0.2)	–8.6% (0.2)
ξ_*v*_	None (–)	NA (–1.0)	–57.4% (0.2)	–50.5% (0.8)	**106.6%** (–0.8)	–22.9% (–0.5)
ϵ_*v*_	None (–)	NA (–1.0)	12.1% (–0.6)	–11.3% (0.6)	22.1% (–0.6)	–7.1% (0.5)
Λ	–18.1% (1.0)	9.3% (–1.0)	1.4% (–0.9)	–2.3% (0.5)	1.7% (–0.4)	0.6% (–0.9)
μ	14.0% (–1.0)	–5.2% (0.8)	–0.3% (0.9)	1.6% (–0.9)	–1.5% (0.9)	–0.0% (0.2)
β_*s*_	–12.3% (0.9)	–49.5% (0.8)	29.0% (–0.4)	**104.5%** (–0.7)	**–80.0%** (0.9)	**51.2%** (–0.5)
β_*a*_	0.1% (–0.1)	–35.7% (0.9)	–1.3% (0.1)	21.1% (0.0)	–30.7% (0.3)	–15.0% (0.2)
η	16.1% (–1.0)	–75.6% (1.0)	36.5% (–0.7)	22.0% (0.4)	77.2% (–0.6)	–19.4% (0.3)
ϕ	41.2% (–1.0)	–19.2% (1.0)	**176.8%** (–1.0)	**–66.4%** (1.0)	13.3% (–0.8)	39.4% (–0.9)
*C*_*s*_	–50.0% (–)	**117.5%** (-)	21.8% (–)	2.8% (–)	-3.7% (–)	36.8% (–)
*C*_*a*_	11.2% (–)	42.3% (–)	-16.9% (–)	2.3% (–)	-4.4% (–)	4.9% (–)

Table 6 summarizes how *R*_0_ and key epidemiological parameters changed between consecutive waves, together with Cliff's Delta values that quantify the magnitude and direction of these shifts. The observed wave-to-wave changes in *R*_0_ align closely with empirical and modeling studies related to SARS-CoV-2 transmission across major variants. The sharp decline between Waves 1 and 2, driven by increased removal of infectious individuals (higher η and ϕ) and reduced symptomatic transmission (lower β_*s*_), is consistent with early-pandemic evidence that strengthened non-pharmaceutical interventions and improved case isolation substantially lowered *R*_0_ (62). The minimal net change from Waves 2 to 3 is analogous to reports that moderate reductions in transmission rates (i.e., decreases in β_*s*_, β_*a*_) and recovery durations (63). The 44.7% drop from Waves 3 to 4 reflects patterns documented during the Delta period, where clinical severity and altered symptomatic proportions interacted with faster recovery in treated individuals (i.e., higher ϕ values) to reduce onward transmission (64). By contrast, the nearly twofold increase between Waves 4 and 5 corresponds to the emergence of Omicron BA.1, whose markedly higher transmissibility (i.e., higher β_*s*_ and β_*a*_ values), faster progression (i.e, higher σ), and increased asymptomatic fraction (i.e., higher *r* values) caused a substantial rise in *R*_0_ (65, 66). The subsequent decline from Waves 5 to 6 shows evidence that widespread vaccination (i.e., higher ξ_*v*_, ϵ_*v*_ values) significantly reduced infectiousness and shortened the infectious period (i.e., higher η, ϕ values) (67). Finally, the moderate rise from Waves 6 to 7 is consistent with the documented immune-evasive properties of XBB lineages, which increased symptomatic infections despite high levels of prior immunity (i.e., higher ω_*v*_ and lower ξ_*v*_, ϵ_*v*_ values) (68, 69). These results demonstrate that the parameter shifts inferred across epidemic waves capture well-established mechanisms of immune waning, variant evolution, and intervention-driven reductions in SARS-CoV-2 transmission supported by the existing literature.

The Table 6 also shows changes in the derived symptomatic and asymptomatic transmission probabilities (*p*_*s*_, *p*_*a*_) and contact rates (*C*_*s*_, *C*_*a*_) across wave transitions. The contact rates *C*_*s*_ and *C*_*a*_ were estimated from social-distancing data reported by Gallup (49), which quantify changes in the average number of close contacts in the United States during the pandemic(see the detailed computation in the Supplementary Section 10). Given these contact rates, we then decomposed the fitted transmission rates into behavioral and biological components via


$$\begin{array}{l} \beta_{s}=C_{s}p_{s},\;\beta_{a}=C_{a}p_{a}. \end{array}$$ (11)

Thus, the infection probabilities *p*_*s*_ and *p*_*a*_ are not separately fitted parameters, but implied by the estimated β_*s*_ and β_*a*_ in combination with the externally informed contact rates. Notably, the increase in *C*_*s*_ and *C*_*a*_ between Wave 3 and Wave 4 aligns with relaxed public health restrictions, while subsequent decreases reflect the reintroduction of containment measures. These parameter trends help explain the shifting transmission dynamics observed across the seven epidemic waves.

The parameters changing patterns are also visually illustrated in Supplementary Figures S2, S3. Supplementary Figure S2 shows how the fitted parameter distributions shift from wave to wave. Supplementary Figures S2a, b indicate that symptomatic transmission (β_*s*_) is highest in Wave 1 and then declines, whereas asymptomatic transmission (β_*a*_) becomes more prominent from Wave 3 onward, consistent with variants that spread more through infections with few or no symptoms; Supplementary Figure S2c shows virulence (δ) elevated in Waves 1 and 7; Supplementary Figures S2d, e illustrate that vaccination rate (ξ_*v*_) and vaccine efficacy (ϵ_*v*_) are low in the early waves, rise to a peak around Wave 5, and then decrease again; and Supplementary Figures S2f, g reveal that symptomatic infection probability (*p*_*s*_) dominates early in the epidemic, whereas asymptomatic infection probability (*p*_*a*_) increases around Waves 3–5. Supplementary Figure S3 summarizes these dynamics in terms of changes between waves. Supplementary Figure S3a shows how vaccination rate (ξ_*v*_) and virulence (δ) often move in opposite directions across transitions, Supplementary Figure S3b depicts joint changes in symptomatic transmission (β_*s*_) and virulence, Supplementary Figure S3c presents the corresponding relationship for asymptomatic transmission (β_*a*_) and virulence, and Supplementary Figure S3d gives boxplots of *R*_0_ for each wave, confirming large drops in *R*_0_ between Waves 1–2, 3–4, and 5–6, a pronounced peak in Wave 5, and a moderate rebound in Wave 7. Together, Table 6 and Supplementary Figures S2, S3 show how changes in transmission, virulence, vaccination, and infection type jointly shape *R*_0_ across the seven waves, highlighting the combined effects of viral evolution and changing control measures.

We also investigated the potential for backward bifurcation across each of the selected time intervals. While several calculated *R*_0_ values were close to the threshold value of 1, none of the fitted parameter sets satisfied the backward-bifurcation condition $\xi_{v}<\xi_{v}^{*}$, where $\xi_{v}^{*}$ is given by Equation 6. This suggests that the likelihood of backward bifurcation was negligible during each of the seven identified peak periods. Consequently, the computed *R*_0_ values were reliable indicators of outbreak dynamics, and disease persistence was unlikely when *R*_0_ < 1.

### 3.5 Posterior probability estimation

The six evolutionary hypotheses are distinguished in our model-based framework by the specific parameter sets they influence and the directional changes observed between epidemic waves. Each hypothesis corresponds to a mechanistic assumption about the evolutionary pressures acting on the pathogen, encoded through changes in transmission rates (β_*s*_, β_*a*_), per-contact infection probabilities (*p*_*s*_, *p*_*a*_), virulence (δ), vaccination parameters (ξ_*v*_, ϵ_*v*_, ω_*v*_), or immunity loss (α). H_1_ (Transmission–Virulence Trade-Off) assumes an inverse relationship between transmission and virulence, where increases in β are accompanied by decreases in δ, whereas H_5_ (Declining Virulence) shares the same parameter set but posits that a sustained decline in δ is independent of any compensatory change in transmission, making it mathematically and conceptually distinct. H_2_ (Short-term Within-host Selection) and H_6_ (Transmission–Virulence Correlation) both involve joint changes in per-contact infection probabilities and virulence: H_2_ represents concurrent increases in infection probabilities and δ reflecting aggressive within-host selection, whereas H_6_ assumes that β-related quantities and δ increase or decrease together without a trade-off. H_3_ (Vaccination-Induced Virulence) and H_4_ (Immune Selection) both represent selection driven by vaccination and immunity, but they act through different mechanisms. H_3_ emphasizes the increase δ under increasing vaccination pressure (ξ_*v*_) and partial protection (ϵ_*v*_ < 1), while H_4_ focuses on changes in the effective loss of protection, represented by the loss of immunity rate α and the rate of decrease in vaccine-induced immunity ω_*v*_ in combination with vaccination (ξ_*v*_). Although our proposed model (Equation 1) does not explicitly distinguish infection progression by vaccination status, so we evaluate H_3_ at the population level: if vaccination coverage increases (higher ξ_*v*_) while protection remains partial (0 < ϵ_*v*_ < 1), H_3_ predicts that selective pressure may favor variants with higher virulence. We basically examine whether waves with higher ξ_*v*_ and incomplete protection coincide with upward shifts in the estimated virulence parameter δ between consecutive waves. In our implementation, immune escape under H_4_ does not require increases in β or δ. Rather, it is supported when epidemic transitions with high population immunity exhibit larger increases in α or ω_*v*_, while any changes in β_*s*_, β_*a*_, or δ are interpreted as secondary effects. Altogether, these hypotheses span trade-offs (H_1_, H_5_), joint increases or decreases in transmission and virulence (H_2_, H_6_), and selection pressures arising from vaccination and waning immunity (H_3_, H_4_). Indeed, the same pattern of parameter changes can be compatible with more than one mechanism (for example, a rise in δ during a highly vaccinated period may be consistent with both H_2_ and H_3_), so some hypotheses can only be partially separated using our data and model structure. As a result, the MBHT framework is used to identify epidemic transitions in which the posterior distributions support a single dominant hypothesis, vs. those in which they indicate joint support for groups of related mechanisms.

To determine the posterior probabilities for competing hypotheses across case transitions, we employed a Bayesian inference framework as the final step in our model-based hypothesis testing (MBHT) approach. This process begins with the estimation of posterior distributions for key epidemiological parameters within each epidemic wave. Specifically, we computed the changes between estimated parameter values of two consecutive waves and formed the dataset *D*. These changes were defined as the differences in the posterior mean of corresponding parameters between waves *W*_*k*_ and *W*_*k*+1_, thus establishing a consistent and interpretable mapping of parameter evolution across transitions.

Using these parameter differences as the basis, we applied kernel density estimation (KDE) to derive the probability density functions (PDFs) for each parameter's change (70). To estimate *P*(*D*|*H*_*i*_), we first generated bootstrapped samples of these differences using posterior draws from each wave. KDE was then applied to these bootstrapped difference samples to form smooth PDFs, representing the likelihood of various change magnitudes under each hypothesis.

For each hypothesis *H*_*i*_, we defined a specific subset of parameters (as in Table 7): *H*_1, set_ = {β_*s*_, β_*a*_, δ}, *H*_2, set_ = {*p*_*s*_, *p*_*a*_, δ}, *H*_3, set_ = {ξ_*v*_, ϵ_*v*_, δ}, *H*_4, set_ = {α, ξ_*v*_, ω_*v*_, δ}, *H*_5, set_ = {β_*s*_, β_*a*_, δ}, and *H*_6, set_ = {*p*_*s*_, *p*_*a*_, δ}. For hypotheses that focus on changes in overall transmissibility and the decline of virulence (H1 and H5), we use the effective transmission rates β_*s*_ and β_*a*_. For hypotheses that emphasize within-host selection or direct transmission–virulence correlations (H2 and H6), we instead use the per-contact infection probabilities *p*_*s*_ and *p*_*a*_, together with fixed contact rates *C*_*s*_ and *C*_*a*_ obtained from social-distancing data reported by Gallup (49). Table 7, we therefore mentioned about *C*_*s*_, *C*_*a*_, *p*_*s*_, , and *p*_*a*_ rather than β_*s*_ and β_*a*_ explicitly. Since we decomposed transmission into a human-controlled component (contact rates *C*_*s*_, *C*_*a*_) and a virus-driven component (infection probabilities *p*_*s*_, *p*_*a*_), so that the effects of behavior and viral properties can be interpreted separately. The effective transmission rates β_*s*_ and β_*a*_ are contained in Table 7 implicitly, as they are simply the products of these contact and infection probabilities. For each set of parameters, we evaluated the KDE-derived PDFs at the observed differences between waves. This provided the likelihood of observing those parameter changes under the given hypothesis.

**Table 7:** Posterior probabilities of six competing mutation hypotheses across seven COVID-19 epidemic wave transitions, highlighting the dominant hypothesis (in bold) for each transition. Here, we used the following sets of parameter values for calculating *P*(*H*_*i*_): *H*_1, set_ = {β_*s*_, β_*a*_, δ}, *H*_2, set_ = {*p*_*s*_, *p*_*a*_, δ}, *H*_3, set_ = {ξ_*v*_, ϵ_*v*_, δ}, *H*_4, set_ = {α, ξ_*v*_, ω_*v*_, δ}, *H*_5, set_ = {β_*s*_, β_*a*_, δ}and*H*_6, set_ = {*p*_*s*_, *p*_*a*_, δ}. The third and fourth rows, respectively, detail the viral effects and human intervention effects and their combined influence on *R*_0_.

**Epidemic wave**	**Wave 1 to 2**	**Wave 2 to 3**	**Wave 3 to 4**	**Wave 4 to 5**	**Wave 5 to 6**	**Wave 6 to 7**
**Posterior probability**	H1: 0.026, **H2: 0.974**	H1: 0.086, H3: 0.029, **H4: 0.886**	H1: 0.031, H2: 0.005, **H4: 0.964**	**H3: 0.445**, H4: 0.083, **H6: 0.471**	H3: 0.099, **H5: 0.885**, H6: 0.017	H1: 0.065, H2: 0.007, H3: 0.298, **H4: 0.630**
**Viral effects**	Δ*δ↑*, Δ*p*_*s*_↑, Δ*p*_*a*_↑	Δ*δ↑*, Δ*α↑*, Δ*p*_*s*_↓, Δ*p*_*a*_↓	Δ*δ↓*, Δ*p*_*a*_↓, Δ*α↑*, Δω_*v*_↑,	Δ*δ↑*, Δ*p*_*s*_↑, Δ*p*_*a*_↑, Δω_*v*_↑	Δ*δ↓*, Δ*p*_*a*_↓, Δ*p*_*s*_↓	Δ*δ↑*, Δ*p*_*s*_↑, Δ*α↑*, Δω_*v*_↑
**Human effects**	Δ*C*_*s*_↓, Δ*C*_*a*_↓, Δ*ϕ↑*, Δ*η↑*	Δξ_*v*_↑, Δϵ_*v*_↑	Δ*C*_*a*_↓, Δ*ϕ↑*, Δ*η↑*, Δϵ_*v*_↑	Δξ_*v*_↓, Δ*C*_*s*_↑, Δ*C*_*a*_↑, Δ*η↑*	Δξ_*v*_↑, Δϵ_*v*_↑, Δ*C*_*s*_↓, Δ*ϕ↑*, Δ*η↑*	Δ*C*_*s*_↑, Δ*C*_*a*_↑, Δξ_*v*_↓
**Compound effect**, ***R*_0_**	Δ*R*_0_↓	Δ*R*_0_↓	Δ*R*_0_↓	Δ*R*_0_↑	Δ*R*_0_↓	Δ*R*_0_↑

After estimating these likelihoods, we computed the joint likelihood for each hypothesis as the product of the individual likelihoods across the selected parameters (71). In conjunction with these likelihoods, we incorporated prior probabilities, which were set to be uniform across hypotheses to reflect no prior preference before analysis. Applying Bayes theorem, we combined the priors and likelihoods to derive the posterior probabilities for each hypothesis:


$$P\left(H_{i}|D_{k}\right)=\frac{P\left(D_{k}|H_{i}\right)P\left(H_{i}\right)}{\sum_{j}P\left(D_{k}|H_{j}\right)P\left(H_{j}\right)}$$

Here, *D*_*k*_ is the set of observed parameter differences between epidemic waves *W*_*k*_ and *W*_*k*+1_ for *k* = 1, …, 6, and *H*_*i*_ (for *i* = 1, …, 6) refers to a specific hypothesis relevant to that transition (see Table 7). *P*(*D*_*k*_|*H*_*i*_) is the likelihood of observing the parameter changes in transition *k* given hypothesis *H*_*i*_, and *P*(*H*_*i*_) is its prior probability.

The posterior probabilities from Table 7 indicate how strongly each hypothesis is supported in different epidemic waves of COVID-19. The mutation from the original strain (Wave 1) to the D614G variant (Wave 2) was best explained by the Short-term within host selection hypothesis; lower probabilities, reflecting their limited role in shaping this transition. Human efforts, including increased vaccination rates (ξ_*v*_) and vaccine efficacy (*epsilon*_*v*_), was able to successfully decrease *R*_0_. In the transition from Wave 3 to Wave 4 (Alpha to Delta Variant), Immune Selection (H4) dominated again with a posterior probability of 0.964, as Delta's mutations enhanced both immune escape and transmissibility. The Short-term within host selection (H2: 0.005) and Transmission-Virulence Trade-Off (H1: 0.031) were less relevant, indicating that Delta's evolutionary trajectory was primarily shaped by immune-driven selection rather than trade-offs or short-term adaptations. Human interventions included reductions in asymptomatic contact rates (*C*_*a*_) and further increases in recovery rates (ϕ, η) and vaccine efficacy (ϵ_*v*_). These measures suppressed *R*_0_, showcasing another human “win” despite the virus's aggressive adaptation.

The transition from Wave 4 to Wave 5 marked the emergence of Omicron BA.1, a variant with complex evolutionary dynamics. The Transmission-Virulence Correlation (H6) had the highest posterior probability (0.471), suggesting a positive relationship between transmissibility and immune escape. Immune Selection (H4: 0.445) also played a significant role, as Omicron BA.1 demonstrated extensive immune evasion. Vaccination-induced virulence (H3: 0.083) was less prominent but relevant, reflecting potential vaccine-driven selective pressure. Despite human interventions such as vaccination (ξ_*v*_) and improved vaccine efficacy (ϵ_*v*_), *R*_0_ increased, marking a virus “win” phase where its rapid adaptation overwhelmed human control efforts. Thereafter, the transition from Omicron BA.1 to BA.2 was dominated by the Declining Virulence hypothesis (H5), with a posterior probability of 0.885. This hypothesis reflects Omicron BA.2's reduced severity while maintaining high transmissibility, suggesting an evolutionary trade-off favoring long-term persistence. The Vaccination-induced virulence (H3: 0.099) and Transmission-Virulence Correlation (H6: 0.017) were less influential, as BA.2's evolutionary strategy prioritized transmissibility over virulence. Human interventions during this phase, including increased vaccination rates (ξ_*v*_), vaccine efficacy (*epsilon*_*v*_), and reduced symptomatic contact rates (*C*_*s*_), successfully suppressed *R*_0_, marking another human “win” phase.

The final transition considered in this study involved the XBB.1.6 variant, with Immune Selection (H4) having the highest posterior probability (0.630), reflecting the variant's advanced immune escape mechanisms. Vaccination-induced virulence (H3: 0.298) was also relevant, indicating the role of vaccine-driven selection in shaping its evolution. Transmission-Virulence Trade-Off (H1: 0.065) and Short-term within host selection (H2: 0.007) had minimal contribution during this transition. Human interventions weakened during this phase, as increased contact rates (*C*_*s*_, *C*_*a*_) and reduced vaccination rates (ξ_*v*_) allowed *R*_0_ to rise, marking a virus “win” phase.

From the above analysis, we conclude that the pandemic was initially governed by Short-term within host selection (H2), while later phases switched to immune escape and transmissibility, as seen with Immune Selection (H4) and Vaccine-Induced Virulence (H3). It also shows that human control measures were effective during early and intermediate transitions, as reflected in reduced *R*_0_ values, but the virus's capacity to adapt through advanced mutation strategies eventually challenged human interventions. This analysis validates the MBHT framework as a robust tool for evaluating and distinguishing between competing hypotheses of pathogen evolution.

## 4 Discussion

The proposed framework addresses the issue of insufficient data to determine the likelihood of competing hypotheses. This framework integrates model formulation, parameter estimation, sensitivity analyses, and available data to capture the dynamic and adaptive interplay between pathogen evolution and public health interventions. Although applied here within the ecological and evolutionary context of infectious diseases, a key strength of the MBHT framework lies in its generalizability to a wide range of complex dynamical systems, where competing hypotheses can be rigorously tested in the case of insufficient data to directly test those hypotheses. When applied to SARS-CoV-2, the framework demonstrates its utility in revealing how evolutionary processes and mitigation efforts jointly influence epidemic trajectories (see Tables 3, 7).

From Figure 3, we can observe that our model captured the sharp peaks and declines observed in waves dominated by the Delta, Omicron BA.1, and Omicron BA.2 variants, respectively. Significant parameter changes between consecutive waves in Table 6 showed that early waves were characterized by sharp reductions in *R*_0_, mainly driven by declines in contact rates (*C*_*s*_, *C*_*a*_) and improvements in recovery rates (η, ϕ). For example, *R*_0_ decreased significantly from wave 1 to wave 2, indicating effective human efforts to suppress viral transmission through social distancing and clinical management. However, during the transition from Wave 4 to Wave 5, *R*_0_ increased markedly, driven by Omicron BA.1's heightened transmissibility and immune escape, reflecting the virus's ability to adapt and overwhelm human control measures. In addition, the LSA identified conditions under which parameters from either the asymptomatic or symptomatic infectious compartments had a stronger effect on *R*_0_. For example, high dominance percentages for β_*a*_ and η in Waves 3 and 5 indicate periods when the spread and recovery patterns of asymptomatic individuals were especially important in shaping transmission potential. Comparison of these dominance patterns with the global sensitivity rankings in Table 5 identifies not only the parameters that are most influential overall, but also the epidemic phases during which targeting them would be most effective in reducing transmission.

A major outcome of the present study is the estimation of posterior probabilities associated with competing hypotheses (see Table 7). The results revealed the dominant evolutionary strategies across wave transitions, linked them to changes in *R*_0_, and revealed the dynamic interplay between viral evolution and human responses from one COVID-19 wave to another. The virus consistently adapted to overcome selective pressures through immune escape and increased transmissibility, while human efforts such as vaccination and reduced contact rates successfully suppressed *R*_0_ during specific phases.

Table 7 results also show that different hypotheses are supported at different waves of the pandemic, rather than a single mechanism explaining all seven waves. In the earliest wave, when almost no one had prior immunity, the patterns were most consistent with short-term within-host selection, where variants that replicate quickly and cause more severe disease can spread before immunity or control measures take effect. As infection- and vaccine-derived immunity build up, the evolution strategy of the variants shifts toward immune selection and vaccination-related pressures, because they can escape existing antibodies or transmit efficiently in partially immune populations. In some transitions, these support a combination of mechanisms rather than a single hypothesis, reflecting the fact that changes in transmissibility, immune escape, and virulence can act together. Thus, our framework does not point to one universal rule for SARS-CoV-2 evolution; instead, it shows that the prevailing evolutionary driver depends on the epidemiological context, and the MBHT approach is generalizable because it can track how the dominant mechanism changes as immunity, vaccination, and control measures evolve over time.

Despite its strengths, our study has some limitations as follows. While we employed a least-squares fitting procedure to estimate epidemic parameters, we acknowledge that a Bayesian inference approach could offer a more principled framework by directly estimating posterior distributions, thereby enhancing the robustness and interpretability of the hypothesis testing process (72, 73). The model assumes homogeneous mixing within the population, which does not account for spatial or demographic heterogeneities. While vaccination and waning immunity were included in the model, variations in vaccine efficacy, reinfection dynamics, and host heterogeneity were simplified. Additionally, the model did not account for the different age groups, or behavioral factors such as vaccine hesitancy. Furthermore, some hypotheses, most notably Vaccination-Induced Virulence (H3), cannot be fully differentiated from related mechanisms such as Immune Selection (H4) or Transmission–Virulence Correlation (H6) as these same set of transmission and immunological parameters. Also, our model does not track strain-specific immune histories. So, immune escape is represented in an aggregate way through the loss-of-immunity parameter α and the vaccine-immunity waning parameter ω_*v*_, which combine shorter-duration same-strain immunity and reduced cross-protection against new variants into a single effective waning process. Future work may incorporate a multi-strain or multi-variant formulation in which individuals carry explicit immune histories, and cross-protection is captured by a structured matrix of immunity levels between strains. We can also extend the model by incorporating age-structured populations, spatial dynamics, and stochastic components to reflect additional host-pathogen complexities better (6, 74–76). Furthermore, as an alternative to the KDE-based estimation of likelihoods, formal statistical techniques such as the likelihood ratio test (77) or Bayes factors (73) could be employed for model comparison.

Beyond COVID-19, the proposed approach offers broad applicability to a range of infectious diseases, including those driven by zoonotic spillovers and rapidly evolving pathogens. It also holds promise for addressing critical global health challenges such as antimicrobial resistance. Future research can further refine and expand the framework to support proactive, data-informed strategies for pandemic preparedness.

Overall, the present study highlights that the dominant evolutionary drivers of SARS-CoV-2 shifted across successive waves in response to changing immunity and intervention measures. Human control measures temporarily reversed increases in *R*_0_ even as the virus continued to adapt. More broadly, the proposed model-based hypothesis testing (MBHT) framework offers a practical and generalizable framework for formalizing, comparing, and interpreting competing evolutionary hypotheses when direct data on underlying mechanisms are limited. In conclusion, this study presents a novel model-based framework for hypothesis testing and understanding the eco-evolutionary dynamics of pathogens under conditions of biological and epidemiological uncertainty.

## Data Availability

The Covid-19 Data was obtained from https://github.com/nytimes/covid-19-data?tab=readme-ov-file. All codes to perform numerical simulations, model fitting and hypothesis testing are available in https://github.com/bsaha1207/barshasaha.

## Footnotes

1 *Daily infected coronavirus cases*. Available online at: https://github.com/nytimes/covid-19-data?tab=readme-ov-file.
