# Supplementary material for: Utility of compartmental models to test the competing hypotheses of pathogen evolution and human intervention
Source: Front Public Health. 2026 Jan 13;13:1702428. doi: 10.3389/fpubh.2025.1702428 (PMC12853639; doi:10.3389/fpubh.2025.1702428)
Supplement: Supplementary file 1 [file Supplementary_file_1.pdf]

Supplementary Document for "Utility of Infectious Disease Models to Test the Competing Hypotheses of Pathogen Evolution and Human Intervention"

Barsha Saha[a] Majid Bani-Yaghoub[a] * Chandranath Podder[b]

[a] Division of Computing, Analytics & Mathematics, School of Science and Engineering, University of Missouri-Kansas City, 5100 Rockhill Rd., Kansas City, Missouri 64110, USA.
[b] Department of Mathematics, University of Dhaka, Nilkhet Rd, Dhaka 1000, Bangladesh.

This supplementary document provides detailed derivations, proofs, analyses, and supporting results referenced in the main manuscript. Its contents are organized as follows.

Contents

1 Proofs of Positivity and Boundedness Theorems 3
2 Derivation of the R_0 Expression 4
3 Proof of Stability Theorems 5
4 Existence of Endemic Equilibrium 9
5 Backward Bifurcation Analysis 13
6 Global Stability Analysis 17
7 Estimated Probability Distributions 19
8 Local Sensitivity Analysis 19
9 Global Sensitivity Analysis 22
10 Estimation of Contact Rates 22
11 Compressions of Changes in Parameter Values 23
12 Goodness of Model Fit 25

*Corresponding author. Email: baniyaghoubm@umkc.edu

List of Figures

S1 *Backward bifurcation of the model (1) with β^* (symptomatic transmission rate) as the bifurcation parameter for Vaccinated $V(t)$ and Symptomatic Infected $I_s(t)$ compartments.* 17

S2 Probability Distribution for Different Parameters across seven waves. **(a-b).** Symptomatic and Asymptomatic Transmission. **(c).** Virulence. **(d).** Vaccination. **(e).** Vaccine Efficacy.**(f-g).**Probability of Symptomatic and Asymptomatic Infection. 20

S3 Percantages of Social Distance Practicing in USA. Source: GALLUP [1] 23

S4 *Visual analysis of key evolutionary and epidemiological traits across COVID-19 case transitions.* ***(a)*** *Dynamics of vaccination and virulence, showing changes in vaccination-induced immunity loss and virulence parameters.* ***(b)*** *Symptomatic transmission-virulence trade-off, illustrating how symptomatic transmission rates evolve in relation to virulence across transitions.* ***(c)*** *Asymptomatic transmission-virulence trade-off, highlighting shifts in asymptomatic transmission rates.* ***(d)*** *Boxplot of reproduction numbers (R_0) for each case, depicting the variability and trends in pathogen transmissibility over the seven epidemic waves.* 24

S5 R-squared (R^2) values across seven consecutive COVID-19 waves 25

List of Tables

S1 Classification performance across seven intervals, showing specificity, sensitivity, and overall accuracy to highlight the model's predictive performance 22

1 Proofs of Positivity and Boundedness Theorems

We formulated our model in the paper as follows:

$$\begin{aligned}
\frac{dS}{dt} &= \Lambda + \omega_v V + \alpha R - \frac{\beta_s I_s + \beta_a I_a}{N} S - (\mu + \xi_v) S \\
\frac{dV}{dt} &= \xi_v S - (1 - \epsilon_v) \frac{\beta_s I_s + \beta_a I_a}{N} V - (\mu + \omega_v) V \\
\frac{dE}{dt} &= \frac{\beta_s I_s + \beta_a I_a}{N} S + (1 - \epsilon_v) \frac{\beta_s I_s + \beta_a I_a}{N} V - (\mu + \sigma) E \\
\frac{dI_a}{dt} &= r\sigma E - (\mu + \eta) I_a \\
\frac{dI_s}{dt} &= (1 - r)\sigma E - (\mu + \delta + \phi) Is \\
\frac{dR}{dt} &= \eta I_a + \phi I_s - (\mu + \alpha) R,
\end{aligned} \tag{1}$$

where all parameters are non-negative constants and the initial conditions of model (1) satisfy the following inequalities:

$$\mathrm{S(0) > 0, V(0) \geq 0, E(0) > 0, I_a(0) > 0, I_s(0) > 0, R(0) \geq 0.} \tag{2}$$

Theorem 1. *(Non-negativity) For all $t > 0$, the solutions $S(t), V(t), E(t), I_a(t), I_s(t), R(t)$ of model (1) with initial condition (2) are non-negative.*

Proof. Let, $t_1 = sup(t > 0 : S > 0, V > 0, E > 0, I_a > 0, I_s > 0, R > 0)$. From the first equation of the model (1), we have

$$\begin{aligned}
\frac{dS}{dt} &= \Lambda + \omega_v V + \alpha R - \frac{\beta_s I_s + \beta_a I_a}{N} S - (\mu + \xi_v) S \\
\frac{dS(t)}{dt} &\geq \Lambda - (\lambda + \mu + \xi_v) S(t), t \in [0, t_1]
\end{aligned} \tag{3}$$

where, $\lambda = \frac{\beta_s I_s + \beta_a I_a}{N}$ is force of infection.

Multiplying both sides of (3) by the integrating factor $\exp\left(\int_0^t \lambda(u)du + (\mu + \xi_v)t\right)$ we get to

$$\frac{d}{dt}\left[S(t)\exp\left(\int_0^t (\lambda(u)du + (\mu + \xi_v)t)\right)\right] \geq \Lambda \exp\left(\int_0^t (\lambda(u)du + \mu + \xi_v)t\right) \tag{4}$$

Solving inequality (4) we obtain,

$$S(t)\exp\left(\int_0^t (\lambda(u)du + (\mu + \xi_v)t)\right) - S(0) \geq \int_0^t \Lambda \exp\left(\int_0^w (\Lambda(u)du + (\mu + \xi_v)t)\right) dw$$

$$\begin{aligned}
S\,(t) \geq& S(0)\exp\left(-\int_0^t (\lambda(u)du + (\mu + \xi_v)t)\right) \\
&+ \exp\left(-\int_0^t (\Lambda(u)du + (\mu + \xi_v)t)\right) \\
&\times \Lambda \int_0^t \exp\left(\int_0^w (\lambda(u)du + (\mu + \xi_v)t)\right) dw > 0,
\end{aligned}$$

Therefore, we obtain $S(t) > 0$ for all $t > 0$. Similarly, we can get $V(t) \geq 0$, $E(t) \geq 0$, $I_a(t) \geq 0$, $I_s(t) \geq 0$ and$R(t) \geq 0$ □

Theorem 2. *(Boundedness) All the solutions of model (1) with initial condition (2) is positively invariant in the feasible region Ω*

Proof. Add all the equation in model (1) to get,

$$\begin{aligned}\frac{dN(t)}{dt} &= \frac{dS}{dt} + \frac{dV}{dt} + \frac{dE}{dt} + \frac{dI_a}{dt} + \frac{dI_s}{dt} + \frac{dR}{dt} \\ &= \Lambda - \mu N - \delta I_s\end{aligned} \tag{5}$$

It is obvious that of $0 < I_s < N$. It follows that,

$$\Lambda - (\mu N + \delta I_s) \leq \frac{dN(t)}{dt} \leq \Lambda - \mu N(t) \tag{6}$$

Thus,

$$\frac{\Lambda}{\mu + \delta} \leq \lim_{t \to \infty} Inf N(t) \leq \lim_{t \to \infty} Sup N(t) \leq \frac{\Lambda}{\mu} \tag{7}$$

This implies,

$$\lim_{t \to \infty} Sup N(t) \leq \frac{\Lambda}{\mu} \tag{8}$$

□

2 Derivation of the R_0 Expression

In this section, we derive the expression for the Reproduction Number R_0 using the next generation matrix approach. Consider the compartments that are related to the infection to obtain the following subsystem,

$$\begin{aligned}\frac{dE}{dt} &= \frac{\beta_s I_s + \beta_a I_a}{N} S + (1 - \epsilon_v) \frac{\beta_s I_s + \beta_a I_a}{N} V - (\mu + \sigma) E \\ \frac{dI_a}{dt} &= r\sigma E - (\mu + \eta) I_a \\ \frac{dI_s}{dt} &= (1 - r)\sigma E - (\mu + \delta + \phi) Is\end{aligned} \tag{9}$$

Let $X = (E, I_a, I_s, S, V, R)$. From the subsystem (9), we arrange transmission vector $\mathscr{F}(X)$ associated with new infection terms and transition vector $\mathscr{V}(X)$ with terms associated with the internal transition of infection given by

$$\mathscr{F}(X) = \begin{bmatrix} \frac{\beta_a I_a + \beta_s I_s}{N} S + (1 - \epsilon_v) \frac{\beta_a I_a + \beta_s I_s}{N} V \\ 0 \\ 0 \end{bmatrix}$$

$$\mathscr{V}(X) = \begin{bmatrix} -(\mu + \sigma) E \\ r\sigma E - (\mu + \eta) I_a \\ (1 - r)\sigma E - (\mu + \delta + \phi) I_s + \eta(1 - \gamma) I_a \end{bmatrix}$$

By taking the Jacobian of $\mathscr{F}(X)$ and $\mathscr{V}(X)$ and evaluating them at the DFE E_{0v}, we get to the non-negative matrix F and the nonsingular matrix V given by

$$F = \begin{bmatrix} 0 & \frac{\beta_a (S^* + (1 - \epsilon_v) V^*)}{N^*} & \frac{\beta_s (S^* + (1 - \epsilon_v) V^*)}{N^*} \\ 0 & 0 & 0 \\ 0 & 0 & 0 \end{bmatrix}$$

$$V = \begin{bmatrix} \mu + \sigma & 0 & 0 \\ -r\sigma & \eta + \mu & 0 \\ -(1 - r)\sigma & 0 & \mu + \delta + \phi \end{bmatrix}$$

Then the basic reproduction number of the model (1) is the spectral radius of the matrix FV^{-1} given by

$$
\begin{aligned}
R_0 &= \rho(FV^{-1}) \\
&= \frac{\sigma(S^* + (1-\epsilon_v)V^*)}{N^*(\mu+\sigma)} \left(\frac{\beta_s(1-r)}{\mu+\delta+\phi} + \frac{r\beta_a}{\mu+\eta} \right) \\
&= \frac{\sigma(\mu+\omega_v+\xi_v(1-\epsilon_v))}{(\mu+\sigma)(\mu+\omega_v+\xi_v)} \left(\frac{r\beta_a}{\mu+\eta} + \frac{(1-r)\beta_s}{\mu+\delta+\phi} \right) \quad \text{(using the values of DFE)} \\
&= \sigma\gamma r R_0^a + \sigma\gamma(1-r)R_0^s
\end{aligned} \tag{10}
$$

where, $\gamma = \frac{\sigma(S^*+(1-\epsilon_v)V^*)}{N^*(\mu+\sigma)}$,which is a function of vaccination, vaccine efficacy, vaccine waning, and mortality rate. Also, $R_0^s = \frac{\beta_s(1-r)}{\mu+\delta+\phi}$ and $R_0^a = \frac{r\beta_a}{\mu+\eta}$

3 Proof of Stability Theorems

Theorem 3. *The DFE, E_{0v}, of our proposed is locally asymptotically stable in the region Ω if $R_{0v} < 1$.*

Proof. Here, the eigenvalues, λ_i (for $i = 1, \dots, 6$) of the Jacobian matrix J that is derived from the system of equations of our model, will be used to analyze the local stability of DFE equation.

The system of equations in the model equation can be written as follows:

$$
\begin{aligned}
f_1 &= \Lambda + \omega_v V + \alpha R - \frac{\beta_s I_s + \beta_a I_a}{N} S - (\mu+\xi_v)S \\
f_2 &= \xi_v S - (1-\epsilon_v)\frac{\beta_s I_s + \beta_a I_a}{N} V - (\mu+\omega_v)V \\
f_3 &= \frac{\beta_s I_s + \beta_a I_a}{N} S + (1-\epsilon_v)\frac{\beta_s I_s + \beta_a I_a}{N} V - (\mu+\sigma)E \\
f_4 &= r\sigma E - (\mu+\eta)I_a \\
f_5 &= (1-r)\sigma E - (\mu+\delta+\phi)Is \\
f_6 &= \eta I_a + \phi I_s - (\mu+\alpha)R
\end{aligned} \tag{11}
$$

Now, we compute the partial derivatives of the right-hand sides of (11) and then construct the Jacobian matrix at the steady state E_{0v} as follows:

$$
J_{DFE} = \begin{bmatrix}
-\mu-\xi_v & \omega_v & 0 & j_{14} & j_{15} & \alpha \\
\xi_v & -\mu-\omega_v & 0 & j_{24} & j_{25} & 0 \\
0 & 0 & -\mu-\sigma & j_{34} & j_{35} & 0 \\
0 & 0 & r\sigma & -\mu-\eta & 0 & 0 \\
0 & 0 & (1-r)\sigma & 0 & -\delta-\mu-\phi & 0 \\
0 & 0 & 0 & \eta & \phi & -\alpha-\mu
\end{bmatrix} \tag{12}
$$

Here,

$$
\begin{aligned}
j_{14} &= \frac{-\beta_a S^*}{N^*} \\
j_{15} &= \frac{-\beta_s S^*}{N^*} \\
j_{24} &= \frac{-\beta_a(1-\epsilon_v)V^*}{N^*} \\
j_{25} &= \frac{-\beta_s(1-\epsilon_v)V^*}{N^*} \\
j_{34} &= \frac{-\beta_a(S^*+(1-\epsilon_v)V^*)}{N^*} \\
j_{35} &= \frac{-\beta_s(S^*+(1-\epsilon_v)V^*)}{N^*}
\end{aligned}
$$

For finding the characteristic polynomial of this matrix, we expand the determinant of characteristic equation $|J-\lambda I|=0$ as follows:

$$|J(E_{0v})-\lambda I| = \begin{vmatrix} -k_1-\lambda & \omega_v & 0 & j_{14} & j_{15} & \alpha \\ \xi_v & -k_2-\lambda & 0 & j_{24} & j_{25} & 0 \\ 0 & 0 & -k_3-\lambda & j_{34} & j_{35} & 0 \\ 0 & 0 & r\sigma & -k_4-\lambda & 0 & 0 \\ 0 & 0 & (1-r)\sigma & 0 & -k_5-\lambda & 0 \\ 0 & 0 & 0 & \eta & \phi & -k_6-\lambda \end{vmatrix} = 0$$

Here,

$$k_1=\mu+\xi_v, k_2=\mu+\omega_v, k_3=\mu+\sigma, k_4=\mu+\eta, k_5=\delta+\mu+\phi, k_6=\alpha+\mu$$

By expanding by the 1st column,

$$\Rightarrow (-k_1-\lambda)\begin{vmatrix} -k_2-\lambda & 0 & \frac{-\beta_a(1-\epsilon_v)V^*}{N^*} & \frac{-\beta_s(1-\epsilon_v)V^*}{N^*} & 0 \\ 0 & -k_3-\lambda & \frac{-\beta_a(S^*+(1-\epsilon_v)V^*)}{N^*} & \frac{-\beta_s(S^*+(1-\epsilon_v)V^*)}{N^*} & 0 \\ 0 & r\sigma & -k_4-\lambda & 0 & 0 \\ 0 & (1-r)\sigma & 0 & -k_5-\lambda & 0 \\ 0 & 0 & \eta & \phi & -k_6-\lambda \end{vmatrix} -$$

$$\xi_v\begin{vmatrix} \omega_v & 0 & -\frac{\beta_a S^*}{N^*} & -\frac{\beta_s S^*}{N^*} & \alpha \\ 0 & -k_3-\lambda & \frac{-\beta_a(S^*+(1-\epsilon_v)V^*)}{N^*} & \frac{-\beta_s(S^*+(1-\epsilon_v)V^*)}{N^*} & 0 \\ 0 & r\sigma & k_4-\lambda & 0 & 0 \\ 0 & (1-r)\sigma & 0 & -k_5-\lambda & 0 \\ 0 & 0 & \eta & \phi & -k_6-\lambda \end{vmatrix} = 0$$

Again expanding by the 1st column,

$$\Rightarrow (-k_1-\lambda)(-k_2-\lambda)\begin{vmatrix} -k_3-\lambda & \frac{-\beta_a(S^*+(1-\epsilon_v)V^*)}{N^*} & \frac{-\beta_s(S^*+(1-\epsilon_v)V^*)}{N^*} & 0 \\ r\sigma & -k_4-\lambda & 0 & 0 \\ (1-r)\sigma & 0 & -k_5-\lambda & 0 \\ 0 & \eta & \phi & -k_6-\lambda \end{vmatrix} -$$

$$\xi_v\omega_v\begin{vmatrix} -k_3-\lambda & \frac{-\beta_a(S^*+(1-\epsilon_v)V^*)}{N^*} & \frac{-\beta_s(S^*+(1-\epsilon_v)V^*)}{N^*} & 0 \\ r\sigma & k_4-\lambda & 0 & 0 \\ (1-r)\sigma & 0 & -k_5-\lambda & 0 \\ 0 & \eta & \phi & k_6-\lambda \end{vmatrix} = 0$$

From above we get,

$$\Rightarrow ((k_1+\lambda)(k_2+\lambda)-\xi_v\omega_v)\begin{vmatrix} -k_3-\lambda & \frac{-\beta_a(S^*+(1-\epsilon_v)V^*)}{N^*} & \frac{-\beta_s(S^*+(1-\epsilon_v)V^*)}{N^*} & 0 \\ r\sigma & -k_4-\lambda & 0 & 0 \\ (1-r)\sigma & 0 & -k_5-\lambda & 0 \\ 0 & \eta & \phi & -k_6-\lambda \end{vmatrix} = 0$$

Finally expanding the above matrix by last column we have,

$$\Rightarrow (\lambda^2+(k_1+k_2)\lambda+(k_2+\xi_v)\mu)(-k_6-\lambda)\begin{vmatrix} -k_3-\lambda & \frac{-\beta_a(S^*+(1-\epsilon_v)V^*)}{N^*} & \frac{-\beta_s(S^*+(1-\epsilon_v)V^*)}{N^*} \\ r\sigma & -k_4-\lambda & 0 \\ (1-r)\sigma & 0 & -k_5-\lambda \end{vmatrix} = 0 \tag{13}$$

Then, from (13) and using the values of k_1, k_2 and k_6 we get,

$$-\alpha - \mu - \lambda = 0$$

$$\Rightarrow \lambda = -(\alpha + \mu) \equiv \lambda_1 \quad \text{(say)} \tag{14}$$

And also,

$$\lambda^2 + \lambda(2\mu + \omega_v + \xi_v) + \mu(\mu + \omega_v + \xi v) = 0$$

$$\begin{aligned}\lambda_{2,3} &= \frac{-(2\mu + \omega_v + \xi_v) \pm \sqrt{(2\mu + \omega_v + \xi_v)^2 - 4\mu(\mu + \omega_v + \xi_v)}}{2} \\ &= \frac{-(2\mu + \omega_v + \xi_v) \pm \sqrt{(\omega_v + \xi_v)^2}}{2}\end{aligned}$$

That is,

$$\lambda_2 = -\mu \quad \text{(for positive)} \tag{15}$$

and,

$$\lambda_3 = -(\mu + \omega_v + \xi_v) \quad \text{(for negative)} \tag{16}$$

The remaining three eigenvalues, we get from the following 3×3 matrix;

$$\begin{vmatrix} -\mu - \lambda & \frac{-\beta_a(S^* + (1-\epsilon_v)V^*)}{N^*} & \frac{-\beta_s(S^* + (1-\epsilon_v)V^*)}{N^*} \\ r\sigma & -\eta - \mu - \lambda & 0 \\ (1-r)\sigma & 0 & -\delta - \mu - \phi - \lambda \end{vmatrix} = 0$$

By expanding above determinant by 3rd column, we get,

$$\begin{aligned}&\frac{-\beta_s\,(S^* + (1-\epsilon_v)V^*)}{N^*}((1-r)\sigma(-\eta - \mu - \lambda)) \\ &+ (-\delta - \mu - \phi - \lambda)[(-\eta - \mu - \lambda)(-\mu - \sigma - \lambda) - \frac{r\sigma\beta_a\,(S^* + (1-\xi_v)V^*)}{N^*} = 0 \\ &\Rightarrow \lambda\left(\frac{(1-r)\sigma\beta_s\,(S^* + (1-\epsilon_v)V^*)}{N^*}\right) + \frac{(1-r)\sigma(\eta+\mu)\beta_s\,(S^* + (1-\epsilon_v V^*))}{N^*} \\ &- (\delta + \mu + \phi + \lambda)(\eta + \mu + \lambda)(\mu + \sigma + \lambda) + \lambda\left(\frac{r\sigma\beta_a\,(S^* + (1-\epsilon_v)V^*)}{N^*}\right) = 0\end{aligned} \tag{17}$$

Now,

$$\begin{aligned}&(\mu + \sigma + \lambda)(\delta + \mu + \phi + \lambda)(\eta + \mu + \lambda) \\ &= (\mu + \sigma + \lambda)(\delta\eta + \delta\mu + \delta\lambda + \mu\eta + \mu^2 + \mu\lambda + \phi\eta + \phi\mu + \phi\lambda + \lambda\eta + \lambda\mu + \lambda^2) \\ &= \lambda^3 + \lambda(\lambda(\delta + 2\mu + \phi + \eta) + (\delta\eta + \delta\mu + \mu\eta + \mu^2 + \phi\eta + \phi\mu)) \\ &+ (\mu + \sigma)(\lambda^2 + \lambda(\delta + 2\mu + \phi + \eta) + (\delta\eta + \delta\mu + \mu\eta + \mu^2 + \phi\eta + \phi\mu)) \\ &= \lambda^3 + \lambda^2(\delta + 3\mu + \sigma + \phi + \eta) + \lambda((\mu + \sigma)(\delta + 2\mu + \phi + \eta) + (\delta + \mu + \phi)(\eta + \mu)) \\ &+ (\mu + \sigma)(\delta + \mu + \phi)(\eta + \mu)\end{aligned} \tag{18}$$

By combining (17) and (18), we get,

$$
\begin{aligned}
&-\lambda^3-\lambda^2(3\mu+\delta+\sigma+\phi+\eta)-\lambda((\mu+\sigma)(\delta+2\mu+\phi+\eta)+(\mu+\delta+\phi)(\mu+\eta))\\
&-(\mu+\sigma)(\mu+\delta+\phi)(\mu+\eta))+\lambda\frac{(1-r)\sigma\beta_s(S^*+(1-\epsilon_v)V^*)}{N^*}+\lambda\frac{r\sigma\beta_a(S^*+(1-\epsilon_v)V^*)}{N^*}\\
&-\left(\frac{(1-r)\sigma(\mu+\eta)\beta_s(S^*+(1-\epsilon_v)V^*)}{N^*}+\frac{r\sigma\beta_a(\mu+\delta+\phi)(S^*+(1-\epsilon_v)V^*)}{N^*}\right)=0\\
&\Rightarrow \lambda^3+\lambda^2(3\mu+\delta+\sigma+\phi+\eta)+\lambda\Bigg((\mu+\sigma)(2\mu+\delta+\phi+\eta)+(\delta+\phi+\eta)(\eta+\mu)\\
&-\frac{(1-r)\sigma\beta_s(S^*+(1-\epsilon_v)V^*)}{N^*}-\frac{r\sigma\beta_a(S^*+(1-\epsilon_v)V^*)}{N^*}\Bigg)+(\mu+\sigma)(\mu+\eta)(\delta+\mu+\phi)\\
&-\left(\frac{(1-r)\sigma(\mu+\eta)\beta_s(S^*+(1-\epsilon_v)V^*)}{N^*}+\frac{r\sigma\beta_a(\delta+\mu+\phi)(S^*+(1-\epsilon_v)V^*)}{N^*}\right)=0
\end{aligned}
\tag{19}
$$

So, the coefficients of above polynomial, are given by,

$$
\begin{aligned}
a_3&=1\\
a_2&=(\delta+\mu+\phi)+(\mu+\sigma)+(\mu+\eta)\\
a_0&=(\mu+\sigma)(\mu+\delta+\phi)(\mu+\eta)-\Bigg(\frac{(1-r)\sigma(\mu+\eta)\beta_s(S^*+(1-\epsilon_v)V^*)}{N^*}\\
&\quad+\frac{r\sigma\beta_a(\delta+\mu+\phi)(S^*+(1-\epsilon_v)V^*)}{N^*}\Bigg)\\
&=(\mu+\sigma)(\mu+\delta+\phi)(\mu+\eta)\Bigg(1-\Bigg(\frac{(1-r)\sigma\beta_s(S^*+(1-\epsilon_v)V^*)}{N^*(\mu+\sigma)(\mu+\delta+\phi)}\\
&\quad+\frac{r\sigma\beta_a(S^*+(1-\epsilon_v)V^*)}{N^*(\mu+\sigma)(\mu+\eta)}\Bigg)\Bigg)\\
&=(\mu+\sigma)(\mu+\delta+\phi)(\mu+\eta)(1-R_{0v})
\end{aligned}
$$

$$
\begin{aligned}
a_1&=(\mu+\eta)\Bigg((\delta+\phi+\mu)+(\mu+\sigma)(\delta+\mu+\phi)+(\mu+\sigma)(\mu+\eta)\\
&-\frac{(1-r)\sigma\beta_s(S^*+(1-\epsilon_v)V^*)}{N^*}-\frac{r\sigma\beta_a(S^*+(1-\epsilon_v)V^*)}{N^*}\Bigg)\\
&=(\mu+\eta)(\delta+\phi+\mu)+(\mu+\sigma)(\delta+\phi+\mu)\left(1-\frac{(1-r)\sigma\beta_s(S^*+(1-\epsilon_v)V^*)}{N^*(\mu+\sigma)(\delta+\phi+\mu)}\right)\\
&+(\mu+\sigma)(\mu+\eta)\left(1-\frac{r\sigma\beta_a(S^*+(1-\epsilon_v)V^*)}{N^*(\mu+\sigma)(\mu+\eta)}\right)\\
&=(\mu+\eta)(\delta+\phi+\mu)+(\mu+\eta)(\delta+\phi+\mu)\Bigg((\mu+\sigma)\\
&-\frac{(\mu+\sigma)(1-r)\sigma\beta_s(S^*+(1-\epsilon_v)V^*)}{N^*(\mu+\sigma)(\mu+\delta+\phi)}+(\mu+\sigma)-\frac{(\mu+\sigma)r\sigma\beta_a(S^*+(1-\epsilon_v)V^*)}{N^*(\mu+\sigma)(\mu+\eta)}\Bigg)\\
&=(\mu+\eta)(\delta+\phi+\mu)(1+2(\mu+\sigma)-(\mu+\sigma)\Bigg(\frac{(1-r)\sigma\beta_s(S^*+(1-\epsilon_v)V^*)}{N^*(\mu+\sigma)(\mu+\delta+\phi)}\\
&+\frac{r\sigma\beta_a(S^*+(1-\epsilon_v)V^*)}{N^*(\mu+\sigma)(\mu+\eta)})\Bigg)\\
&=(\mu+\eta)(\delta+\phi+\mu)(1+(\mu+\sigma)+(\mu+\sigma)(1-R_{0v}))
\end{aligned}
$$

Clearly, a_3, $a_2>0$ and $a_1>0$, (strictly positive) if $(1-R_{0v})>0$ or, if $R_{0v}<1$. We know, by the Routh-Hurwitz criterion, above polynomial have negative roots if $a_2>0$, $a_1>0$, $a_0>0$ and also if $a_2a_1>a_0$,

$(a_2a_1 - a_0 > 0)$. Now,

$$\begin{aligned}
&(\delta+\mu+\phi)+(\mu+\sigma)+(\mu+\eta)(\mu+\eta)(\delta+\phi+\mu)\\
&(1+(\mu+\sigma)+(\mu+\sigma)(1-R_{0v}))>(\mu+\sigma)(\mu+\delta+\phi)(\mu+\eta)(1-R_{0v})\\
&\text{By simplification we get,}\\
&\Rightarrow(3\mu+\delta+\sigma+\phi+\eta)+(3\mu+\delta+\sigma+\phi+\eta)(\mu+\sigma)+(\mu+\sigma)(3\mu+\sigma+\\
&\eta+\phi+\delta)(1-R_{0v})-(\mu+\sigma)(1-R_{0v})>0
\end{aligned}\tag{20}$$

It is clear, this inequality holds if $(1 - R_{0v}) > 0$ or $R_{0v} < 1$. So, the disease free equilibrium (DFE), E_{0v} is locally asymptotically stable (LAS) when $R_{0v} < 1$. □

4 Existence of Endemic Equilibrium

We can find the conditions for the existence of endemic equilibrium as follows. Let the endemic equilibrium point of our proposed model be,
$E_{1v} = (S^{**}, V^{**}, E^{**}, I_a^{**}, I_s^{**}, R^{**})$
and at the endemic equilibrium (EE), the expressions this becomes,

$$\begin{aligned}
&\Lambda+\omega_vV^{**}+\alpha R^{**}-\frac{\beta_sI_s^{**}+\beta_aI_a^{**}}{N^{**}}S^{**}-k1S^{**}=0\\
&\xi_vS^{**}-k2\frac{\beta_sI_s^{**}+\beta_aI_a^{**}}{N^{**}}V^{**}-k3V^{**}=0\\
&\frac{\beta_sI_s^{**}+\beta_aI_a^{**}}{N^{**}}S^{**}+k2\frac{\beta_sI_s^{**}+\beta_aI_a^{**}}{N^{**}}V^{**}-k4E^{**}=0\\
&r\sigma E^{**}-k5I_a^{**}=0\\
&k6\sigma E^{**}-k7Is^{**}=0\\
&\eta I_a^{**}+\phi I_s^{**}-k8R^{**}=0
\end{aligned}\tag{21}$$

where,$k1 = (\mu + \xi_v), k2 = (1 - \epsilon_v), k3 = (\mu + \omega_v), k4 = (\mu + \sigma)$,
$k5 = (\mu + \eta), k6 = (1 - r), k7 = (\mu + \delta + \phi), k8 = (\mu + \alpha) and,$
$N^{**}(t) = S^{**}(t) + V^{**}(t) + E^{**}(t) + I_a^{**}(t) + I_s^{**}(t) + R^{**}(t)$
force of infection,$\lambda^* = \frac{\beta_sI_s^{**}+\beta_aI_a^{**}}{N^{**}}$

By solving the above equations and simplifying them, we get the following implicit form of Endemic Equilibrium values:

$$\begin{aligned}
S^{**}&=\frac{k4(\lambda^*k2+k3)E^{**}}{\lambda^{*2}k2+\lambda^*(k3+\xi_vk2)}\\
V^{**}&=\frac{\xi_vk4E^{**}}{\lambda^{*2}k2+\lambda^*(k3+\xi_vk2)}\\
I_a^{**}&=\frac{r\sigma E^{**}}{k5}\\
I_s^{**}&=\frac{k6\sigma E^{**}}{k7}\\
R^{**}&=\frac{(k7\eta r\sigma+k5k6\phi\sigma)E^{**}}{k5k7k8}
\end{aligned}\tag{22}$$

Now,putting these values in, $\lambda^* = \frac{\beta_sI_s^{**}+\beta_aI_a^{**}}{N^{**}}$

$$\Rightarrow\lambda^*=\frac{(\sigma\beta_s(1-r)(\mu+\eta)+\sigma\beta_ar(\mu+\delta+\phi))E^{**}}{N^{**}(\mu+\eta)(\mu+\delta+\phi}$$

$$\Rightarrow N^{**}(\mu+\eta)(\mu+\delta+\phi)\lambda^{*} = (\sigma\beta_s(1-r)(\mu+\eta)+\sigma\beta_a r(\mu+\delta+\phi))E^{**} \tag{23}$$

$$\text{Let,}\psi_1 = \lambda^{*2}k2+\lambda^{*}(k3+\xi_v k2) = \lambda^{*2}(1-\epsilon_v)+\lambda^{*}((\mu+\omega_v)+\xi_v(1-\epsilon_v) \tag{24}$$

$$\begin{aligned}\text{Now,}N^{**} &= S^{**}+V^{**}+E^{**}+I_a^{**}+I_s^{**}+R^{**}\\ &= \Bigg(\frac{(\mu+\sigma)(\lambda^{*}(1-\xi_v)+(\mu+\omega_v))}{\psi_1}+\frac{\xi_v(\mu+\sigma)}{\psi_1}+1+\\ &\frac{r\sigma}{(\mu+\eta)}+\frac{(1-r)\sigma}{(\mu+\delta+\phi)}+\frac{\sigma\eta r(\mu+\delta+\phi)+\phi\sigma(1-r)(\mu+\eta)}{(\mu+\eta)(\mu+\delta+\phi)(\mu+\alpha)}\Bigg)E^{**}\end{aligned} \tag{25}$$

After some calculation,from above equation (25),we get,

$$N^{**} = \frac{\begin{aligned}&(\lambda^{*}(\mu+\sigma)(\mu+\eta)(\mu+\delta+\phi)(\mu+\alpha)(1-\xi_v)+(\mu+\sigma)(\mu+\eta)(\mu+\delta+\phi)\\ &(\mu+\alpha)(\mu+\omega_v)+\xi_v(\mu+\sigma)(\mu+\eta)(\mu+\delta+\phi)(\mu+\alpha)+r\sigma\psi_1(\mu+\delta+\phi)(\mu+\alpha)\\ &+(1-r)\sigma\psi1(\mu+\eta)(\mu+\alpha)+\sigma\eta r\psi_1(\mu+\delta+\phi)+\phi\sigma(1-r)\psi_1(\mu+\eta)\\ &+\psi_1(\mu+\eta)(\mu+\delta+\phi)(\mu+\alpha))E^{**}\end{aligned}}{(\mu+\eta)(\mu+\delta+\phi)(\mu+\alpha)\psi_1}$$

putting this value of N^{**} in equation (23) and divide both side by E^{**} (since $E^{**}>0$),we have,

$$\begin{aligned}&(\mu+\eta)(\mu+\delta+\phi)\lambda^{*}\frac{\begin{aligned}&(\lambda^{*}(\mu+\sigma)(\mu+\eta)(\mu+\delta+\phi)(\mu+\alpha)(1-\xi_v)+(\mu+\sigma)(\mu+\eta)\\ &(\mu+\delta+\phi)(\mu+\alpha)(\mu+\omega_v)+\xi_v(\mu+\sigma)(\mu+\eta)(\mu+\delta+\phi)(\mu+\alpha)\\ &+r\sigma\psi_1(\mu+\delta+\phi)(\mu+\alpha)+(1-r)\sigma\psi_1(\mu+\eta)(\mu+\alpha)+\\ &\sigma\eta r\psi_1(\mu+\delta+\phi)+\phi\sigma(1-r)\psi_1(\mu+\eta)+\psi(\mu+\eta)\\ &(\mu+\delta+\phi)(\mu+\alpha))E^{**}\end{aligned}}{(\mu+\eta)(\mu+\delta+\phi)(\mu+\alpha)\psi_1}\\ &=\sigma\beta_s(1-r)(\mu+\eta)+\sigma\beta_a r(\mu+\delta+\phi)\end{aligned}$$

$$\begin{aligned}&\Rightarrow\lambda^{*}(\lambda^{*}(\mu+\sigma)(\mu+\eta)(\mu+\delta+\phi)(\mu+\alpha)(1-\xi_v)+(\mu+\sigma)(\mu+\eta)\\ &(\mu+\delta+\phi)(\mu+\alpha)(\mu+\omega_v)+\xi_v(\mu+\sigma)(\mu+\eta)(\mu+\delta+\phi)(\mu+\alpha)\\ &+r\sigma\psi_1(\mu+\delta+\phi)(\mu+\alpha)+(1-r)\sigma\psi_1(\mu+\eta)(\mu+\alpha)+\sigma\eta r\psi_1(\mu+\delta+\phi)\\ &+\phi\sigma(1-r)\psi_1(\mu+\eta)+\psi_1(\mu+\eta)(\mu+\delta+\phi)(\mu+\alpha))\\ &=(\sigma\beta_s(1-r)(\mu+\eta)+\sigma\beta_a r(\mu+\delta+\phi))(\mu+\alpha)\psi_1\end{aligned} \tag{26}$$

Now, substituting the value of ψ_1 in above (26) equation,we get,

$$
\begin{aligned}
\Rightarrow&\lambda^*(\lambda^*(\mu+\sigma)(\mu+\eta)(\mu+\delta+\phi)(\mu+\alpha)(1-\xi_v)+(\mu+\sigma)(\mu+\eta)\\
&(\mu+\delta+\phi)(\mu+\alpha)(\mu+\omega_v)+\xi_v(\mu+\sigma)(\mu+\eta)(\mu+\delta+\phi)(\mu+\alpha)\\
&+\lambda^{*2}r\sigma(\mu+\delta+\phi)(\mu+\alpha)(1-\epsilon_v)+\lambda^*((\mu+\omega_v)+\xi_v(1-\epsilon_v))r\sigma(\mu+\delta+\phi)(\mu+\alpha)\\
&+\lambda^{*2}(1-r)\sigma(\mu+\eta)(\mu+\alpha)(1-\epsilon_v)+\lambda^*((\mu+\omega_v)+\xi_v(1-\epsilon_v))(1-r)\sigma(\mu+\eta)(\mu+\alpha)\\
&+\lambda^{*2}\sigma\eta r(\mu+\delta+\phi)(1-\epsilon_v)+\lambda^*((\mu+\omega_v)+\xi_v(1-\epsilon_v))\sigma\eta r(\mu+\delta+\phi)+\\
&\lambda^{*2}\phi\sigma(1-r)(\mu+\eta)(1-\epsilon_v)+\lambda^*((\mu+\omega_v)+\xi_v(1-\epsilon_v))\phi\sigma(1-r)(\mu+\eta)+\\
&\lambda^{*2}(\mu+\eta)(\mu+\delta+\phi)(\mu+\alpha)(1-\epsilon_v)+\lambda^*((\mu+\omega_v)+\xi_v(1-\epsilon_v))(\mu+\eta)(\mu+\delta+\phi)(\mu+\alpha))\\
&=\lambda^{*2}\sigma\beta_s(1-r)(\mu+\eta)(\mu+\alpha)(1-\epsilon_v)+\lambda^*((\mu+\omega_v)+\xi_v(1-\epsilon_v))\sigma\beta_s(1-r)(\mu+\eta)\\
&(\mu+\alpha)+\lambda^{*2}\sigma\beta_a r(\mu+\delta+\phi)(\mu+\alpha)(1-\epsilon_v)+\lambda^*((\mu+\omega_v)+\xi_v(1-\epsilon_v))\sigma\beta_a r\\
&(\mu+\delta+\phi)(\mu+\alpha)
\end{aligned}
\tag{27}
$$

Now,dividing both side by λ^*,we get,

$$
\begin{aligned}
\Rightarrow&\lambda^{*2}((1-\epsilon_v)(r\sigma(\mu+\delta+\phi)(\mu+\alpha)+(1-r)\sigma(\mu+\eta)(\mu+\alpha)+\sigma\eta r(\mu+\delta+\phi)+\\
&\phi\sigma(1-r)(\mu+\eta)+(\mu+\eta)(\mu+\delta+\phi)(\mu+\alpha)))+\lambda^*((1-\epsilon_v)((\mu+\sigma)(\mu+\eta)\\
&(\mu+\delta+\phi)(\mu+\alpha)-\sigma\beta_s(1-r)(\mu+\eta)(\mu+\alpha)-\sigma\beta_a r(\mu+\delta+\phi)(\mu+\alpha))+\\
&((\mu+\omega_v)+\xi_v(1-\epsilon_v))(r\sigma(\mu+\delta+\phi)(\mu+\alpha)+(1-r)\sigma(\mu+\eta)(\mu+\alpha)+\\
&\sigma\eta r(\mu+\delta+\phi)+\phi\sigma(1-r)(\mu+\eta)+(\mu+\eta)(\mu+\delta+\phi)(\mu+\alpha)))+(\mu+\sigma)(\mu+\eta)\\
&(\mu+\delta+\phi)(\mu+\alpha)(\mu+\omega_v)+\xi_v(\mu+\sigma)(\mu+\eta)(\mu+\delta+\phi)(\mu+\alpha)-((\sigma\beta_a r\\
&(\mu+\delta+\phi)(\mu+\alpha)+\sigma\beta_s(1-r)(\mu+\eta)(\mu+\alpha))((\mu+\omega_v)+\xi_v(1-\epsilon_v)))=0
\end{aligned}
\tag{28}
$$

the above (28) has the following quadratic form:

$$
a_2\lambda^{*2}+a_1\lambda^*+a_0=0 \tag{29}
$$

By some simplification, we get the coefficients of (29) as follows:

$$
\begin{aligned}
a_0&=(\mu+\sigma)(\mu+\eta)(\mu+\delta+\phi)(\mu+\alpha)(\mu+\omega_v+\xi_v)-(\sigma\beta_a r(\mu+\delta+\phi)\\
&(\mu+\alpha)+\sigma\beta_s(1-r)(\mu+\eta)(\mu+\alpha))(\mu+\omega_v+\xi_v-\xi_v\epsilon_v)\\
&=(\mu+\sigma)(\mu+\eta)(\mu+\delta+\phi)(\mu+\alpha)(\mu+\omega_v+\xi_v)\left(1-\frac{r\sigma\beta_a(\mu+\omega_v+\xi_v(1-\epsilon_v))}{(\mu+\sigma)(\mu+\eta)}\right.\\
&\left.-\frac{(1-r)\sigma\beta_s(\mu+\omega_v+\xi_v(1-\epsilon_v))}{(\mu+\sigma)(\mu+\delta+\phi)}\right)\\
&=(\mu+\sigma)(\mu+\eta)(\mu+\delta+\phi)(\mu+\alpha)(\mu+\omega_v+\xi_v)(1-R_{0v})
\end{aligned}
$$

$$
\begin{aligned}
a_1 &= (1-\epsilon_v)((\mu+\sigma)(\mu+\eta)(\mu+\delta+\phi)(\mu+\alpha) - \sigma\beta_s(1-r)(\mu+\eta)(\mu+\alpha) \\
&- \sigma\beta_a r(\mu+\delta+\phi)(\mu+\alpha)) + (\mu+\omega_v+\xi_v(1-\epsilon_v))(r\sigma(\mu+\delta+\phi)(\mu+\alpha) \\
&+ (1-r)\sigma(\mu+\eta)(\mu+\alpha) + \sigma\eta r(\mu+\delta+\phi) + \phi\sigma(1-r)(\mu+\eta)+ \\
&(\mu+\eta)(\mu+\delta+\phi)(\mu+\alpha)) \\
&= (\mu+\sigma)(\mu+\eta)(\mu+\delta+\phi)(\mu+\alpha)(1-\epsilon_v)\Bigg(1 - \frac{r\sigma\beta_a}{(\mu+\sigma)(\mu+\eta)} \\
&- \frac{(1-r)\sigma\beta_s}{(\mu+\sigma)(\mu+\delta+\phi)}\Bigg) + (\mu+\omega_v+\xi_v(1-\epsilon_v))(r\sigma(\mu+\delta+\phi)(\mu+\alpha) \\
&+ \sigma(\mu+\eta)(\mu+\alpha) - \sigma(\mu+\eta)(\mu+\alpha) + \sigma\eta r(\mu+\delta+\phi) + \phi\sigma(\mu+\eta) \\
&- r\phi\sigma(\mu+\eta) + (\mu+\eta)(\mu+\delta+\phi)(\mu+\alpha)) \\
&= (\mu+\sigma)(\mu+\eta)(\mu+\delta+\phi)(\mu+\alpha)(1-\epsilon_v)(1-R_0) + (\mu+\omega_v+ \\
&\xi_v(1-\epsilon_v))(r\sigma(\mu+\delta+\phi)(\mu+\alpha+\eta) + \sigma(\mu+\eta)(\mu+\alpha+\phi) - r\sigma \\
&(\mu+\alpha+\phi)(\mu+\eta) + (\mu+\eta)(\mu+\delta+\phi)(\mu+\alpha)) \\
&= (\mu+\eta)(((\mu+\alpha)(\mu+\delta+\phi) + \sigma(\mu+\alpha+\phi)(1-r)\sigma\frac{(\mu+\delta+\phi)(\mu+\alpha+\eta)}{(\mu+\eta)}) \\
&(\mu+\omega_v+\xi_v(1-\epsilon_v)) - (\mu+\sigma)(\mu+\delta+\phi)(\mu+\alpha)(1-\epsilon_v)(1-R_0)) \\
&= (\mu+\eta)(((\mu+\alpha)(\mu+\delta+\phi) + \sigma(\mu+\alpha+\phi)(1-r)\sigma b \\
&(\mu+\omega_v+\xi_v(1-\epsilon_v)) - (\mu+\sigma)(\mu+\delta+\phi)(\mu+\alpha)(1-\epsilon_v)(1-R_0))
\end{aligned}
$$

where,b=$\frac{(\mu+\delta+\phi)(\mu+\alpha+\eta)}{(\mu+\eta)}$

$$
\begin{aligned}
a_2 &= (1-\epsilon_v)(r\sigma(\mu+\delta+\phi)(\mu+\alpha) + (1-r)\sigma(\mu+\eta)(\mu+\alpha) + \sigma\eta r(\mu+\delta+\phi)+ \\
&\phi\sigma(1-r)(\mu+\eta) + (\mu+\eta)(\mu+\delta+\phi)(\mu+\alpha)) \\
&= (\mu+\eta)(\mu+\alpha)(\mu+\delta+\phi) + \sigma(\mu+\alpha+\phi)(1-r)\sigma b)(1-\epsilon_v)
\end{aligned}
$$

The quadratic equations (29) can be studied to identify the possible multiple endemic equilibria for $R_{0v} < 1$.It should be noted that the coefficient a_2 is always positive, and a_0 is negative if $R_{0v} > 1$.The sign of a_1 can determine the possibility of multiple equilibria and the bifurcation phenomenon. The results can be summarized as follows

Theorem 4. *Our proposed COVID-19 model with vaccination has*

i. A unique endemic equilibrium if $a_0 < 0 \iff R_{0v} > 1$.
ii. A unique endemic equilibrium if $a_1 < 0$ *and* $a_0 = 0$ *or the discriminant,* $\triangle = a_1^2 - 4a_0a_2 = 0$.
iii.Two endemic equilibrium if $a_0 > 0, a_1 < 0$ *and* $a_1^2 - 4a_0a_2 > 0$.
iv. no endemic equilibrium otherwise.(i.e. $a_1 > 0$ *and* $a_0 > 0$.
This theorem confirms that the model does not have any endemic equilibrium when $R_{0v} < 1$*. Thus, the model does not show backward bifurcation when there is no reinfection.*

Proof. We have noted earlier that the coefficient a_2 is always positive, and a_0 is negative if $R_{0v} > 1$.For case (i) where $a_0 < 0$ (that is,$R_{0v} > 1$) an unique endemic equilibrium exists since there is only one change of sign according to Descarte's Rule of Signs.

for case (ii) where $a_1 < 0$ and $R_{0v} = 1 (i.e. a_0 = 0)$, the above quadratic equation,(3.38) reduces to $a_2\lambda^* + a_1 = 0$ and in this case, the model equation will have a unique positive endemic equilibrium if $a_1 < 0$ and no positive non-trivial equilibrium if $a_1 \geq 0$.

For case (iii) where,$a_1 < 0$ and $R_{0v} < 1 (i.e. a_0 > 0)$,there is exactly two changes of sign by **Descartes** law indicating the existence of two non-trivial positive equilibrium.

For case (iv) where $a_1 > 0$ and $R_{0v} < 1(i.e. a_0 > 0)$,then there are no changes of signs.Thus, there is no endemic equilibrium in such a case.

Hence, from case(iii), it can be concluded that the model equation (3.1) have a maximum of two endemic equilibrium when $R_{0v} < 1, a_1 < 0$ and $a_1^2 - 4a_0a_2 > 0$.

□

5 Backward Bifurcation Analysis

Theorem 5. *Let,*

$$\xi_v^* = \frac{1 + (\mu + \omega_v)(w_4 + w_5 + w_6)}{(w_1\epsilon_v - (1 - \epsilon_v)(w_4 + w_5 + w_6)(\mu + \omega_v)w_2 + (1 - \epsilon_v))}$$

Then, at $R_{0v} = 1$, our model system exhibits transcritical(backward) bifurcation and its direction is backward iff $\xi_v > \xi_v^*$

Proof. Suppose that $E_{1v} = (S^{**}, V^{**}, E^{**}, I_a^{**}, I_s^{**}, R^{**})$ represents any arbitrary endemic equilibrium of our model (that is, an equilibrium in which at least one of the infected components is nonzero). Applying the Center Manifold theory, the existence of backward bifurcation will be studied. Let,$S = x_1, V = x_2, E = x_3, I_a = x_4, I_s = x_5, R = x_6$,so that $N = x_1 + x_2 + x_3 + x_4 + x_5 + x_6$.Further, by using the vector notation $X = (x_1, x_2, x_3, x_4, x_5, x_6)^T$, the model can be written in the form $\frac{dX}{dt} = F(X)$, with $(f_1, f_2, f_3, f_4, f_5, f_6)^T$, as follows:

$$\begin{aligned}
\frac{dx_1}{dt} &= f_1 = \Lambda + \omega_v x_2 + \alpha x_6 - \frac{\beta_s x_5 + \beta_a x_4}{N} S - (\mu + \xi_v)x_1 \\
\frac{dx_2}{dt} &= f_2 = \xi_v x_1 - (1 - \epsilon_v)\frac{\beta_s x_5 + \beta_a x_4}{N} V - (\mu + \omega_v)x_2 \\
\frac{dx_3}{dt} &= f_3 = \frac{\beta_s x_3 + \beta_a x_4}{N} S + (1 - \epsilon_v)\frac{\beta_s x_5 + \beta_a x_4}{N} V - (\mu + \sigma)x_3 \\
\frac{dx_4}{dt} &= f_4 = r\sigma x_3 - (\mu + \eta)x_4 \\
\frac{dx_5}{dt} &= f_5 = (1 - r)\sigma x_3 - (\mu + \delta + \phi)x_5 \\
\frac{dx_6}{dt} &= f_6 = \eta x_4 + \phi x_5 - (\mu + \alpha)x_6
\end{aligned} \tag{30}$$

Hence,the force of infection becomes, $\lambda = \frac{\beta_s x_5 + \beta_a x_4}{N}$
Considering the case when $R_{0v} = 1$ and $\beta_s = \beta^*$ is chosen as a bifurcation parameter.Solving for $\beta_s = \beta_*$ in $R_{0v} = 1$, we get,

$$1 = \frac{\sigma(\mu + \omega_v + \xi_v(1 - \epsilon_v))(r\beta_a(\mu + \delta + \phi) + (1 - r)\beta_*(\mu + \eta))}{(\mu + \sigma)(\mu + \omega_v + \xi_v)}$$

So,

$$\beta_* = \frac{(\mu + \sigma)(\mu + \omega_v + \xi_v) - \sigma(\mu + \omega_v + \xi_v(1 - \epsilon_v))r\beta_a(\mu + \delta + \phi)}{\sigma(\mu + \omega_v + \xi_v(1 - \epsilon_v)(1 - r)(\mu + \eta)}$$

since,we let earlier,$k1 = (\mu + \xi_v), k2 = (1 - \epsilon_v), k3 = (\mu + \omega_v), k4 = (\mu + \sigma)$,
$k5 = (\mu + \eta), k6 = (1 - r), k7 = (\mu + \delta + \phi), k8 = (\mu + \alpha)$ and $k9 = (\mu + \omega_v + \xi_v)$
Then,

$$\beta_* = \frac{k_4(k_1 + k_3 - \mu) - \sigma(k_3 + k_2\xi_v r\beta_a k_7}{\sigma(k_1 + k_3 - \mu)k_5k_6}$$

So, the linearization matrix of the above system around the DFE E_{0v} is given by calculating the Jacobian

for the transformed model (30) as follows:

$$J^*_{E_{0v}}|_{\beta_s=\beta*} = \begin{bmatrix} -k_1 & \omega_v & 0 & \frac{-\beta_a k_3}{k_9} & -\beta_s k_3 k_9 & \alpha \\ \xi_v & -k_3 & 0 & \frac{-\beta_a k_2 \xi_v}{k_9} & \frac{-\beta_s k_2 \xi_v}{k_9} & 0 \\ 0 & 0 & -k_4 & \frac{\beta_a(k_3+k_2\xi_v)}{k_9} & \frac{\beta_a(k_3+k_2\xi_v)}{k_9} & 0 \\ 0 & 0 & r\sigma & -k_5 & 0 & 0 \\ 0 & 0 & k_6\sigma & 0 & -k_7 & 0 \\ 0 & 0 & 0 & \eta & \phi & -k_8 \end{bmatrix} \tag{31}$$

The Jacobian $J^*_{E_{0v}}$ of (30) with $\beta_s = \beta*$ has a simple zero eigenvalue (with all other eigenvalues having negative real part). Hence, the center manifold theory can be used to analyze the dynamics of the model. So, the right (w) and left(v) eigenvectors corresponding to zero eigenvalues are computed from the above matrix (31) as follows:

Right eigenvector: $J^*_{E_{0v}}\underline{w} = \underline{0}$
where,

$$\underline{w} = \begin{pmatrix} w_1 \\ w_2 \\ w_3 \\ w_4 \\ w_5 \\ w_6 \end{pmatrix} \quad \text{and} \quad \underline{0} = \begin{pmatrix} 0 \\ 0 \\ 0 \\ 0 \\ 0 \\ 0 \end{pmatrix}.$$

So, for eigenvalue zero, we get the following system from above $J^*_{E_{0v}}\underline{w} = \underline{0}$ as follows:

$$\begin{aligned} &-k_1 w_1 + \omega_v w_2 - \frac{\beta_a k_3}{k_9} w_4 - \frac{\beta^* k_3}{k_9} w_5 + \alpha w_6 = 0 \\ &\xi_v w_1 - k_3 w_2 - \frac{\beta_a k_2 \xi_v}{k_9} w_4 - \frac{\beta_* k_2 \xi_v}{k_9} w_5 = 0 \\ &-k_4 w_3 + \frac{\beta a(k_3 + k_2\xi_v)}{k_9} w_4 + \frac{\beta*(k_3 + k_2\xi_v)}{k_9} w_5 = 0 \\ &r\sigma w_3 - k_5 w_4 = 0 \\ &k_6\sigma w_3 - k_7 w_5 = 0 \\ &\eta w_4 + \phi w_5 - k_8 w_6 = 0 \end{aligned} \tag{32}$$

By solving the above equations we get the right eigenvector as follows:

$$\begin{aligned} w_1 =& \frac{k_3}{\xi_v} w_2 + \frac{k_2\sigma(r\beta_a k_7 + \beta^* k_5 k_6)}{k_5 k_7 k_9} w_3 \\ w_2 =& \frac{\begin{array}{c}\xi_v(k_1 k_2 k_8 \sigma(\beta_a r k_7 + \beta^* k_5 k_6) + k_3 k_7 k_8 \beta_a r\sigma + \\ \beta^* k_3 k_5 k_6 k_8 \sigma - \alpha\eta r\sigma k_7 k_9 - \alpha\phi\sigma k_5 k_6 k_9)\end{array}}{k_5 k_7 k_8 k_9(\omega_v \xi_v - k + 3)} w_3 \\ w_3 =& w_3 > 0 \\ w_4 =& \frac{r\sigma w_3}{k_5} \\ w_5 =& \frac{\sigma k_6 w_3}{k_7} \\ w_6 =& \frac{\eta r\sigma k_7 + \phi\sigma k_5 k_6}{k_5 k_7 k_8} w_3 \end{aligned} \tag{33}$$

Similarly, we'll get the left eigenvector.

Left Eigenvector: $\underline{v}J^*_{E_{0v}} = \underline{0}$,
where

$$\underline{v} = \begin{pmatrix} v_1 & v_2 & v_3 & v_4 & v_5 & v6 \end{pmatrix} \quad \text{and} \quad \underline{0} = \begin{pmatrix} 0 & 0 & 0 & 0 & 0 & 0 \end{pmatrix}.$$

So, for eigenvalue zero we get the following system from above $\underline{v}J^*_{E_{0v}} = \underline{0}$ as follows:

$$\begin{aligned}
& -k_1 v_1 + \xi_v v_2 = 0 \\
& \omega_v v_1 - k_3 v_2 = 0 \\
& -k_4 v_3 + r\sigma v_4 + \sigma k_6 v_5 = 0 \\
& \frac{-\beta_a k_3}{k_9} v_1 - \frac{\beta_a k_2 \xi_v}{k_9} v_2 + \frac{\beta_a (k_3 + k_2 \xi_v)}{k_9} v_3 - k_5 v_4 + \eta v_6 = 0 \\
& \frac{-\beta^* k_3}{k_9} v_1 - \frac{\beta^* k_2 \xi_v}{k_9} v_2 + \frac{\beta^* (k_3 + k_2 \xi_v)}{k_9} v_3 - k_7 v_5 + \phi v_6 = 0 \\
& -\alpha v_1 - k_8 v_6 = 0
\end{aligned} \tag{34}$$

By solving these equations, we get the left eigenvectors as follows:

$$\begin{aligned}
v_1 =& 0 \\
v_2 =& 0 \\
v_3 =& v_3 > 0 \\
v_4 =& \frac{\beta_a (k_3 + k_2 \xi_v)}{k_5 k_9} \\
v_5 =& \frac{\beta^* (k_3 + k_2 \xi_v)}{k_7 k_9} \\
v_6 =& 0
\end{aligned} \tag{35}$$

Furthermore, the associated non-zero-second partial derivatives of model (30) evaluated at (E_{ov}, β^*) is obtained as:

$$\begin{aligned}
\frac{\partial f_3}{\partial x_1 \partial x_4} &= \frac{\partial f_3}{\partial x_4 \partial x_1} = \frac{\beta_a \mu \xi_v \epsilon_v}{\Lambda(\mu + \omega_v + \xi_v)} \\
\frac{\partial f_3}{\partial x_1 \partial x_5} &= \frac{\partial f_3}{\partial x_5 \partial x_1} = \frac{\beta^* \mu \xi_v \epsilon_v}{\Lambda(\mu + \omega_v + \xi_v)} \\
\frac{\partial f_3}{\partial x_2 \partial x_4} &= \frac{\partial f_3}{\partial x_4 \partial x_2} = \frac{-\beta_a \mu \epsilon_v (\mu + \omega_v)}{\Lambda(\mu + \omega_v + \xi_v)} \\
\frac{\partial f_3}{\partial x_2 \partial x_5} &= \frac{\partial f_3}{\partial x_5 \partial x_2} = \frac{-\beta^* \mu \epsilon_v (\mu + \omega_v)}{\Lambda(\mu + \omega_v + \xi_v)} \\
\frac{\partial f_3}{\partial x_3 \partial x_4} &= \frac{\partial f_3}{\partial x_4 \partial x_3} = \frac{-\beta_a \mu (\mu + \omega_v + (1 - \epsilon_v)\xi_v)}{\Lambda(\mu + \omega_v + \xi_v)} \\
\frac{\partial f_3}{\partial x_3 \partial x_5} &= \frac{\partial f_3}{\partial x_5 \partial x_3} = \frac{-\beta^* \mu (\mu + \omega_v + (1 - \epsilon_v)\xi_v)}{\Lambda(\mu + \omega_v + \xi_v)} \\
\frac{\partial f_3}{\partial x_4 \partial x_4} &= \frac{-2\mu \beta_a (\mu + \omega_v + (1 - \epsilon_v)\xi_v)}{\Lambda(\mu + \omega_v + \xi_v)} \\
\frac{\partial f_3}{\partial x_4 \partial x_5} &= \frac{\partial f_3}{\partial x_5 \partial x_4} = \frac{-\mu(\beta_a + \beta_*)(\mu + \omega_v + (1 - \epsilon_v)\xi_v)}{\Lambda(\mu + \omega_v + \xi_v)} \\
\frac{\partial f_3}{\partial x_4 \partial x_6} &= \frac{\partial f_3}{\partial x_6 \partial x_4} = \frac{-\mu \beta_a (\mu + \omega_v + (1 - \epsilon_v)\xi_v)}{\Lambda(\mu + \omega_v + \xi_v)} \\
\frac{\partial f_3}{\partial x_5 \partial x_5} &= \frac{-2\mu \beta^* (\mu + \omega_v + (1 - \epsilon_v)\xi_v)}{\Lambda(\mu + \omega_v + \xi_v)} \\
\frac{\partial f_3}{\partial x_5 \partial x_6} &= \frac{\partial f_3}{\partial x_6 \partial x_5} = \frac{\beta^* \mu (\mu + \omega_v + (1 - \epsilon_v)\xi_v)}{\Lambda(\mu + \omega_v + \xi_v)}
\end{aligned}$$

Now, the coefficient of $\bar{a}$ and $\bar{b}$ from center manifold theorem is calculated as follows:

Here.we take w_3 and v_3 equal to 1.

$$\begin{aligned}
\bar{b} &= \sum_{k,i=1}^{6} v_k w_i \frac{\partial^2 f_k}{\partial x_i \partial \beta}(E_{0v}, 0) \\
&= v_1 w_4 \frac{\partial^2 f_1}{\partial x_1 \partial \beta} + v_1 w_5 \frac{\partial^2 f_1}{\partial x_5 \partial \beta} + v_2 w_4 \frac{\partial^2 f_2}{\partial x_4 \partial \beta} \\
&\quad + v_2 w_5 \frac{\partial^2 f_2}{\partial x_5 \partial \beta} + v_3 w_4 \frac{\partial^2 f_3}{\partial x_4 \partial \beta} + v_3 w_5 \frac{\partial^2 f_3}{\partial x_5 \partial \beta} \\
&= 0 + 0 + 0 + 0 + 0 + \frac{\sigma(1-r)(\mu + \omega_v + (1-\epsilon_v)\xi_v v_3)}{(\mu + \omega_v + \xi_v)(\mu + \delta + \phi)} w_3 \\
&= \frac{\sigma(1-r)(\mu + \omega_v + (1-\epsilon_v)\xi_v}{(\mu + \omega_v + \xi_v)(\mu + \delta + \phi)} > 0 \; [since 0 < \epsilon_v < 1 \text{ and } 0 < r < 1]
\end{aligned} \tag{36}$$

$$\begin{aligned}
\bar{a} &= \sum_{k,j,=1}^{6} v_k w_i w_j \frac{\partial^2 f_k}{\partial x_i \partial \beta}(E_0, \beta^*) \\
&= v_3 w_1 \left[w_4 \frac{\partial f_3}{\partial x_1 \partial x_4} + w_5 \frac{\partial f_3}{\partial x_1 \partial x_5} \right] + v_3 w_2 \left[w_4 \frac{\partial f_3}{\partial x_2 \partial x_4} + w_5 \frac{\partial f_3}{\partial x_2 \partial x_5} \right] \\
&\quad + v_3 w_4 \left[w_1 \frac{\partial f_3}{\partial x_4 \partial x_1} + w_2 \frac{\partial f_3}{\partial x_4 \partial x_2} + w_3 \frac{\partial f_3}{\partial x_4 \partial x_3} + w_4 \frac{\partial f_3}{\partial x_4 \partial x_4} \right. \\
&\quad \left. + w_5 \frac{\partial f_3}{\partial x_4 \partial x_5} + w_6 \frac{\partial f_3}{\partial x_4 \partial x_6} \right] + v_3 w_5 \left[w_1 \frac{\partial f_3}{\partial x_5 \partial x_1} + w_2 \frac{\partial f_3}{\partial x_5 \partial x_2} \right. \\
&\quad \left. + w_3 \frac{\partial f_3}{\partial x_5 \partial x_3} + w_4 \frac{\partial f_3}{\partial x_5 \partial x_4} + w_5 \frac{\partial f_3}{\partial x_5 \partial x_5} + w_6 \frac{\partial f_3}{\partial x_5 \partial x_6} \right] \\
&\quad + v_3 w_3 \left[w_4 \frac{\partial f_3}{\partial x_3 \partial x_4} + w_5 \frac{\partial f_3}{\partial x_3 \partial x_5} \right] + v_3 w_6 \left[w_4 \frac{\partial f_3}{\partial x_6 \partial x_4} + w_5 \frac{\partial f_3}{\partial x_6 \partial x_5} \right]
\end{aligned} \tag{37}$$

$$\bar{a} = \frac{\begin{aligned} &2\mu(\beta_a w_4 + \beta^* w_5)(-\mu\epsilon_v w_2 - \epsilon_v \omega_v w_2 + \xi_v \epsilon_v w_1 + \xi_v \epsilon_v w_4 + \xi_v \epsilon_v w_5 \\ &+ \xi_v \epsilon_v w_6 - \mu w_4 - \mu w_5 - \mu w_6 + \xi_v \epsilon_v - \omega_v w_4 - \omega_v w_5 - \omega_v w_6 \\ &- \xi_v w_4 - \xi_v w_5 - \xi_v w_6 - \mu - \omega_v - \xi_v) \end{aligned}}{\Lambda(\mu + \omega_v + \xi_v)}$$

After some simplification we get the above $\bar{a}$ value as,

$$\bar{a} = \frac{\begin{aligned} &2\mu(\beta_a w_4 + \beta^* w_5)(\xi_v \epsilon_v w_1 - (\mu + \omega_v + (1-\epsilon_v)\xi_v)(w_4 + w_5 + w_6) \\ &- 2\mu(\beta_a w_4 + \beta^* w_5)((\mu + \omega_v)(\xi_v w_2 + (1-\epsilon_v) + 1)) \end{aligned}}{\Lambda(\mu + \omega_v + \xi_v)}$$

By centre manifold theorem the model (30) exhibits Backward Bifurcation iff $\bar{a} > 0$,so from above equation after simplifying the value for ξ_v,we get our desired condition,that is:

$$\xi_v > \frac{1 + (\mu + \omega_v)(w_4 + w_5 + w_6)}{(w_1 \epsilon_v - (1-\epsilon_v)(w_4 + w_5 + w_6)(\mu + \omega_v)w_2 + (1-\epsilon_v))}$$

Based on the computed coefficient of $\bar{a}$ and $\bar{b}$, it is clear that coefficient $\bar{b}$ defined in equation (37) is always positive. Therefore, model system (30) will undergoes backward bifurcation at $R_{0V} = 1$, if $\bar{a} > 0$, that is if $(\xi_v^* < \xi_v)$ and will undergo a forward bifurcation at $R_{0V} = 1$, if $\bar{a} < 0$, that is if $(\rho^* > \rho)$, conclusively, this establish theorem. □

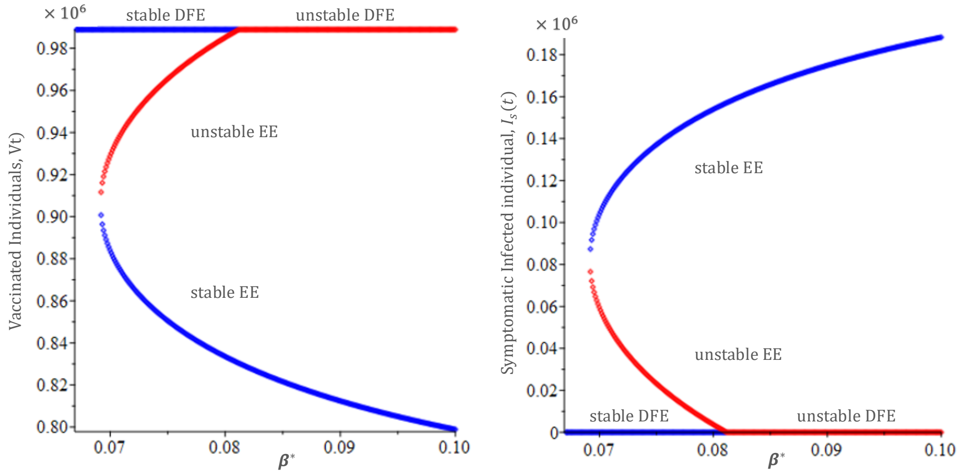

Figure S1: *Backward bifurcation of the model (1) with β^* (symptomatic transmission rate) as the bifurcation parameter for Vaccinated $V(t)$ and Symptomatic Infected $I_s(t)$ compartments.*

Figure S1 illustrates the dynamics of the backward bifurcation in the vaccinated compartments (V) and symptomatic infected (I_s) compartments, with β^* (symptomatic transmission rate) used as the bifurcation parameter. As β^* increases, the system transitions from a stable disease-free equilibrium to a bistable region, where both disease-free and endemic equilibria coexist. This behavior indicates that backward bifurcation occurs when β^* interacts with key parameters that govern vaccination and transmission dynamics. Higher vaccination rates reduce effective transmission (β^*), driving the system toward a disease-free equilibrium. Conversely, lower vaccination rates allow β^* to remain high, sustaining endemic equilibria. Thus, the condition of backward bifurcation clearly shows the importance of vaccination.

6 Global Stability Analysis

Theorem 6. *Let $\epsilon_v = 1$ and $R_0 < 1$, then the DFE of model (1) is globally asymptotically stable in Ω.*

Proof. To show this, we define the Lyapunov function as follows:

$$L = P_1 E + P_2 I_a + P_3 I_s \tag{38}$$

where,

$$P_1 = (\mu + \eta)(\mu + \delta + \phi)$$
$$P_2 = \beta_a(\mu + \delta + \phi)$$
$$P_3 = \beta_s(\mu + \eta)$$

The time differential of L gives,

$$
\begin{aligned}
\frac{dL}{dt} &= P_1\frac{dE}{dt} + P_2\frac{dI_a}{dt} + P_3\frac{dI_s}{dt} \\
&= P_1\left(\frac{\beta_a I_a + \beta_s I_s}{N}S - (\mu+\sigma)E\right) + P_2\left(r\sigma E - (\mu+\eta)I_a\right) + P_3\left((1-r)\sigma E\right. \\
&\left. - (\mu+\delta+\phi)I_s\right) \\
&= \left(P_1\frac{\beta_a S}{N} - P_2(\mu+\eta)\right)I_a + \left(P_1\frac{\beta_s S}{N} - P_3(\mu+\delta+\phi)\right)I_s + \left(P_3(1-r)\sigma + P_2 r\sigma\right. \\
&\left. - P_1(\mu+\sigma)\right)E \\
&= \left(P_1\frac{\beta_a S}{N} - P_2(\mu+\eta)\right)I_a + \left(P_1\frac{\beta_s S}{N} - P_3(\mu+\delta+\phi)\right)I_s + (\beta_s(1-r)\sigma(\mu+\eta) \\
&+ \beta_a r\sigma(\mu+\delta+\phi) - (\mu+\eta)(\mu+\delta+\phi)(\mu+\sigma))E
\end{aligned}
$$

Note that $S(t) \leq N(t)$, for every $t \in \Omega$ and then we have,

$$\frac{dL}{dt} \leq ((\mu+\sigma)(\mu+\eta)(\mu+\delta+\phi)(R_0 - 1)) < 0$$

Hence, by the Lasalle's invariance principal, the DFE of model 1 is GAS in Ω if $R_0 < 1$. □

The following theorem will establish the conditions for the global stability of EE without re-infection and with complete vaccine efficacy.

Theorem 7. *If $R_0 > 1$, vaccine efficacy $\epsilon_v = 1$ and the re-infection terms, $\omega_v = \alpha = 0$, then the EE, $E_{1v} = (S^{**}, V^{**}, E^{**}, I_a^{**}, I_s^{**}, R^{**})$ with $\lambda = \frac{\beta_a I_a^{**} + \beta_s I_s^{**}}{N^{**}}$, is globally asymptotically stable.*

Proof. Let,

$$\frac{\beta_a}{N^{**}} = \frac{\beta_a \mu}{\Lambda} = \beta_1', \quad \frac{\beta_s}{N^{**}} = \frac{\beta_s \mu}{\Lambda} = \beta_2'$$

Consider the following Lyapunov function of the Goh-Volterra type:

$$
\begin{aligned}
V =& S - S^{**} - ln\frac{S}{S^{**}} + E - E^{**} - ln\frac{E}{E^{**}} + \\
& a_1(I_a - I_a^{**} - ln\frac{I_a}{I_a^{**}}) + a_2(I_s - I_s^{**} - ln\frac{I_s}{I_s^{**}})
\end{aligned}
\tag{39}
$$

where a_1 and a_2 are positive constants to be determined. Now, differentiate (39) with respect to t,

$$\dot{V} = (1 - \frac{S^{**}}{S})\dot{S} + (1 - \frac{E^{**}}{E})\dot{E} + a_1(1 - \frac{I_a^{**}}{I_a})\dot{I}_a + a_2(1 - \frac{I_s^{**}}{I_s})\dot{I}_s$$

Substituting the values of $\dot{S}, \dot{E}, \dot{I}_a$ and $\dot{I}_s$ from system (1) and noted that at steady state,

$$\Lambda = (\frac{\beta_a I_a^{**} + \beta_s I_s^{**}}{N^{**}})S^{**} + \mu S^{**} + \xi_v S^{**}$$

i.e.

$$\Lambda = (\beta_1' I_a^{**} + \beta_2' I_s^{**})S^{**} + \mu S^{**} + \xi_v S^{**}$$

Then we get,

$$\dot{V} = (1 - \frac{S^{**}}{S})(\xi_v S^{**} + \mu S^{**} + (\beta_1' I_a^{**} + \beta_2' I_s^{**})S^{**} - \xi_v S - \mu S - (\beta_1' I_a + \beta_2' I_s)S)$$
$$+ (\beta_1' I_a + \beta_2' I_s)S - k_4 E - ((\beta_1' I_a + \beta_2' I_s)S)\frac{E_{**}}{E} + k_4 E^{**}$$
$$+ \frac{(\beta_1' I_a^{**} + \beta_2' I_s^{**})S^{**}}{k_5 I_a^{**}}(r\sigma E - k_5 I_a - r\sigma E\frac{I_a^{**}}{I_a} + k_5 I_a^{**})$$
$$+ \frac{(\beta_1' I_a^{**} + \beta_2' I_s^{**})S^{**}}{k_7 I_s^{**}}(k_6\sigma E - k_7 I_s - k_6\sigma E\frac{I_s^{**}}{I_s} + k_7 I_s^{**})$$

Here,

$$k_4 = \mu + \sigma, k_5 = \mu + \eta, k_6 = 1 - r, k_7 = \mu + \delta + \phi, r\sigma = \frac{k_5 I_a^{**}}{E^{**}}, (1-r)\sigma = \frac{k_7 I_s^{**}}{E^{**}},$$
$$a_1 = \frac{(\beta_1' I_a^{**} + \beta_2' I_s^{**})S^{**}}{k_5 I_a^{**}}, a_2 = \frac{(\beta_1' I_a^{**} + \beta_2' I_s^{**})S^{**}}{k_7 I_s^{**}}$$

Now, by some simplifications, we have,

$$\dot{V} = (\mu + \xi_v)S^{**}(2 - \frac{S^{**}}{S} - \frac{S}{S^{**}}) + \beta_1' I_a^{**} S^{**}(4 - \frac{S^{**}}{S} - \frac{E^{**} S I_a}{E S^{**} I_a^{**}} - \frac{I_a^{**} E}{I_a E^{**}} - \frac{I_s}{I_s^{**}})$$
$$+ \beta_2' I_s^{**} S^{**}(4 - \frac{S^{**}}{S} - \frac{E^{**} S I_s}{E S^{**} I_s^{**}} - \frac{I_s^{**} E}{I_s E^{**}} - \frac{I_a}{I_a^{**}})$$

Since the arithmetic mean exceeds the geometric mean, it follows that,

$$2 - \frac{S^{**}}{S} - \frac{S}{S^{**}} \leq 0$$
$$4 - \frac{S^{**}}{S} - \frac{E^{**} S I_a}{E S^{**} I_a^{**}} - \frac{I_a^{**} E}{I_a E^{**}} - \frac{I_s}{I_s^{**}} \leq 0$$
$$4 - \frac{S^{**}}{S} - \frac{E^{**} S I_s}{E S^{**} I_s^{**}} - \frac{I_s^{**} E}{I_s E^{**}} - \frac{I_a}{I_a^{**}} \leq 0$$

Therefore, we have $\dot{V} \leq 0$ for $R_0 > 1$. Thus, by the Lyapunov function V and LaSalle's Invariance principle, every solution to the model (1) approaches the EE, E_{1v} as $t \to \infty$, for $R_0 > 1$ □

7 Estimated Probability Distributions

As explained in the paper, we estimated the probability distributions of model parameters using the spline method. Figure S2 illustrates the changing dynamics of parameters driving the transmission and evolution of SARS-CoV-2 across seven epidemic waves. The probability density estimations of symptomatic and asymptomatic transmission rates (β_s and β_a), virulence rates (δ), vaccination rates (ξ_v), and probabilities of symptomatic and asymptomatic infections (p_s and p_a) provide insights into how the virus adapted over time in response to public health measures and evolving immunity. These distributions were created using a statistical interpolation technique that applies piecewise polynomial smoothing, similar to a spline fit. This method transforms histogram data of parameter values into continuous probability density functions, ensuring smoothness and continuity. These density curves allow for detailed comparisons of parameter trends and their influence on the reproduction number (R_0), forming a key basis for hypothesis testing in this study.

8 Local Sensitivity Analysis

Local (elasticity) sensitivity analysis quantifies the relative change in the basic reproduction number R_0 with respect to a given parameter. This is valuable in disease modeling because it allows us to rank which

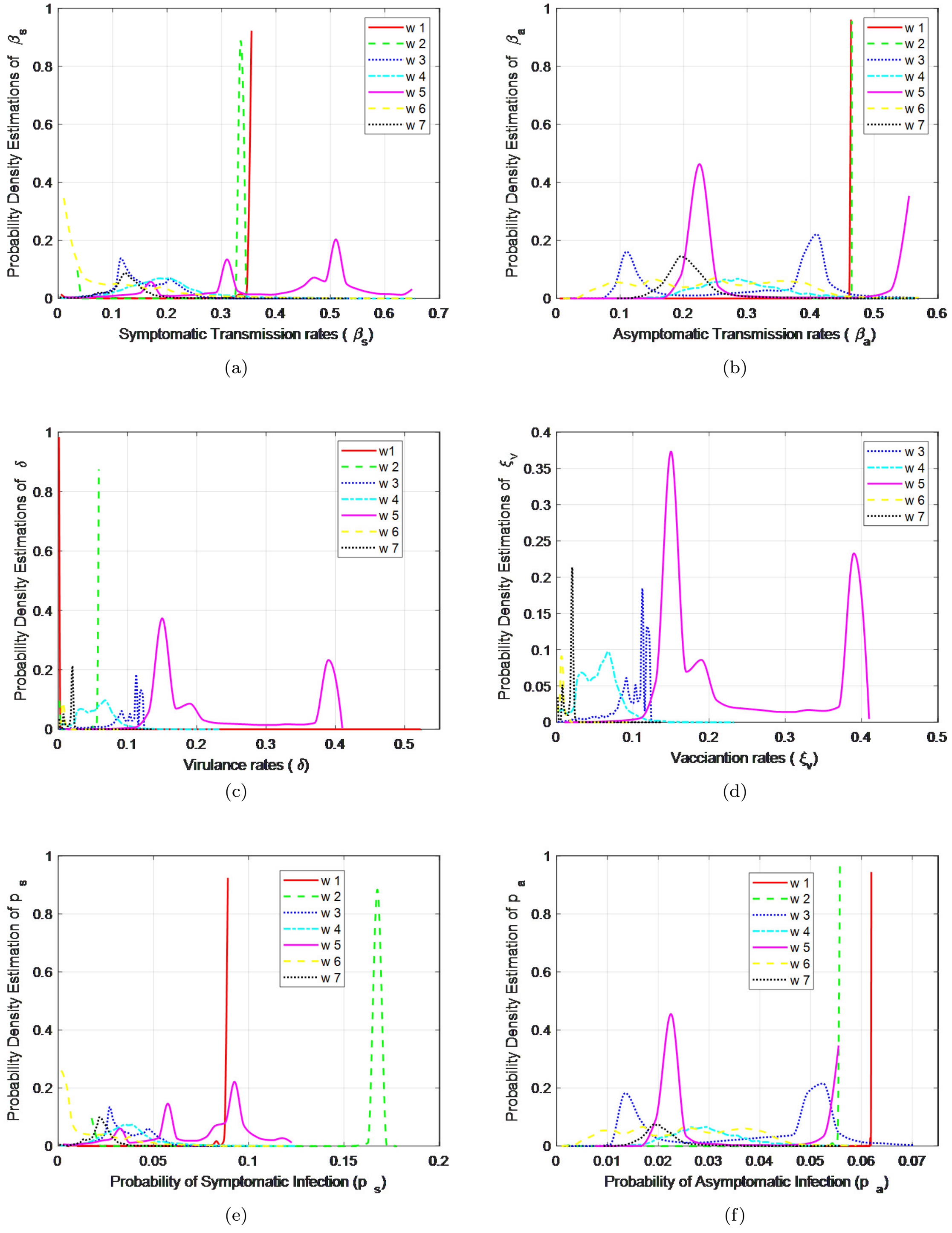

Figure S2: Probability Distribution for Different Parameters across seven waves. **(a-b).** Symptomatic and Asymptomatic Transmission. **(c).** Virulence. **(d).** Vaccination. **(e).** Vaccine Efficacy.**(f-g).**Probability of Symptomatic and Asymptomatic Infection.

parameters have the greatest proportional effect on transmission potential, and therefore, which control

measures may be most effective. The elasticity index for a parameter ω is defined as

$$\Gamma_{R_0}^{\omega} = \frac{\partial R_0}{\partial \omega}\frac{\omega}{R_0}. \tag{40}$$

The sign of $\Gamma_{R_0}^{\omega}$ shows whether R_0 increases or decreases with ω, and its magnitude ranks the relative importance of ω in driving R_0.

Theorem 8 (Transmission-rate dominance)**.** *The asymptomatic transmission rate β_a has a larger elasticity than the symptomatic rate β_s if and only if*

$$\frac{\beta_a}{\beta_s} > \frac{1-r}{r}\frac{\mu+\eta}{\mu+\delta+\phi}. \tag{41}$$

i.e., a proportional increase in β_a raise R_0 more than the same increase in β_s, indicating asymptomatic transmission becomes the more influential target for intervention when this condition holds.

Proof. From (10),

$$\frac{\partial R_0}{\partial \beta_a} = \gamma\frac{r}{\mu+\eta}, \qquad \frac{\partial R_0}{\partial \beta_s} = \gamma\frac{1-r}{\mu+\delta+\phi}.$$

Using (40),

$$\Gamma_{R_0}^{\beta_a} = \gamma\frac{r}{\mu+\eta}\frac{\beta_a}{R_0}, \qquad \Gamma_{R_0}^{\beta_s} = \gamma\frac{1-r}{\mu+\delta+\phi}\frac{\beta_s}{R_0}.$$

Thus,

$$\frac{\Gamma_{R_0}^{\beta_a}}{\Gamma_{R_0}^{\beta_s}} = \frac{r}{1-r}\frac{\beta_a}{\beta_s}\frac{\mu+\delta+\phi}{\mu+\eta} > 1$$

The inequality $\Gamma_{R_0}^{\beta_a} > \Gamma_{R_0}^{\beta_s}$ holds exactly when (41) is satisfied, completing the proof. When the inequality become reverse, symptomatic transmissition rate, β_s become more influential than the asymptomatic transmission rate β_a. Lastly, both parameters have same elasticity index if the condition becomes equal. □

Remark 1. When, $r \to 0$, symptomatic transmission always dominates. But when, $r \to 1$, asymptomatic transmission always dominates, regardless of other parameters.

Theorem 9 (Removal-rate dominance)**.** *The elasticity of the asymptomatic removal rate η is greater than that of the symptomatic removal rate ϕ if and only if*

$$\frac{\eta}{\phi} > \frac{1-r}{r}\frac{\beta_s}{\beta_a}\left(\frac{\mu+\eta}{\mu+\delta+\phi}\right)^2. \tag{42}$$

i.e., this means that increasing η by a fixed proportion would reduce R_0 more than the same proportional increase in ϕ.

Proof. Differentiating (10):

$$\frac{\partial R_0}{\partial \eta} = -\gamma\, r\frac{\beta_a}{(\mu+\eta)^2}, \qquad \frac{\partial R_0}{\partial \phi} = -\gamma\,(1-r)\frac{\beta_s}{(\mu+\delta+\phi)^2}.$$

From (40),

$$\Gamma_{R_0}^{\eta} = -\gamma\, r\frac{\beta_a}{(\mu+\eta)^2}\frac{\eta}{R_0}, \qquad \Gamma_{R_0}^{\phi} = -\gamma\,(1-r)\frac{\beta_s}{(\mu+\delta+\phi)^2}\frac{\phi}{R_0}.$$

Taking their ratio:

$$\frac{\Gamma_{R_0}^{\eta}}{\Gamma_{R_0}^{\phi}} = \frac{r}{1-r}\frac{\beta_a}{\beta_s}\frac{\eta}{\phi}\left(\frac{\mu+\delta+\phi}{\mu+\eta}\right)^2.$$

The inequality $\Gamma_{R_0}^{\eta} > \Gamma_{R_0}^{\phi}$ holds exactly when (42) is satisfied. □

Remark 2. When, $r \to 0$, symptomatic removal dominates unless η/ϕ is extremely large. But $r \to 1$, asymptomatic removal always dominates.

9 Global Sensitivity Analysis

We use the machine learning models of Classification and Regression Tree (CRT) to assess the global sensitivity of model parameters with respect to the basic reproduction number R_0. The following Table S1 evaluates the CRT model's predictive performance that we have used to do the sensitivity analysis.

Table S1: Classification performance across seven intervals, showing specificity, sensitivity, and overall accuracy to highlight the model's predictive performance

**Cases**	**Specificity (no)**	**Sensitivity (yes)**	**Overall**
Interval 1	97.5%	99.9%	99.2%
Interval 2	99.8%	95.5%	99.4%
Interval 3	98.6%	72.5%	92.4%
Interval 4	98.3%	83.8%	96.4%
Interval 5	97.3%	89.4%	95.5%
Interval 6	99.6%	93.7%	98.9%
Interval 7	99.0%	85.2%	97.5%

Table S1 evaluates the CRT model's ability to distinguish between extreme and non-extreme R_0 values. The model achieved high overall accuracy, exceeding 95% in all but one interval, demonstrating its robustness in identifying influential parameters. In particular, sensitivity and specificity values remained consistently high across intervals, highlighting the model's reliability. The performance during specific intervals, such as the first and sixth, where overall accuracies were 99.2% and 98.9%, respectively, underscores the CRT approach's effectiveness in capturing dynamic changes in parameter importance as the pandemic progressed.

10 Estimation of Contact Rates

In our modeling framework, we decomposed the symptomatic and asymptomatic transmission rates (β_s, β_a) as products of human contact rates (C_s, C_a) with infectious individuals and the corresponding per-contact infection probabilities (p_s, p_a). In particular, $\beta_s = C_s \cdot p_s$ and $\beta_a = C_a \cdot p_a$.
This decomposition separates behavioral factors (captured by contact rates) from biological factors (captured by infection probabilities) in the transmission process. The transmission rates (β_s, β_a) were fitted independently for each of the seven epidemic waves in our dataset. Each wave corresponds to the peak period of a distinct SARS-CoV-2 variant in the United States. In contrast, we estimated the contact rates (C_s, C_a) using external behavioral data from Gallup [1], which reports social distancing practices in the United States during the pandemic (see Figure S3).

The Gallup survey documented three categories of distancing behavior. For our calculations, we focused on the percentage of people avoiding public places (e.g., stores and restaurants), represented by the green curve in Figure S3, from early 2020 through January 2022. During the height of the pandemic, small gatherings and large events (e.g., concerts and sporting events) were restricted by government lockdowns, making avoidance of public places the most reliable indicator of daily contact behavior. Additionally, a rapid review of social contact patterns during COVID-19 [2] found that individuals had approximately 2–5 contacts per day during the pandemic, compared to pre-COVID rates of 7–26 contacts per day.

Using these observations, we estimated contact rates by assuming a proportional relationship between the percentage of people not avoiding public places and the corresponding effective daily contact range for the pre-COVID and during the COVID:

$$\frac{\text{Percentage of people not avoiding public places in pre-COVID}}{\text{Percentage of people not avoiding public places during COVID}} = \frac{\text{Pre-COVID contact range}}{\text{Contact range during COVID, } C}. \tag{43}$$

We used pre-COVID contact ranges of 7 contacts/day for symptomatic individuals and 26 contacts/day for asymptomatic individuals, consistent with [2]. To illustrate the calculation, consider Wave 1 (21 January 2020–21 June 2020). From Figure S3, approximately 79% of people avoided public places at the Wave 1 peak, so 21% were not avoiding them. For the pre-COVID baseline, we use Gallup's post-January-2022 value, approximately 20% avoidance (meaning 80% not avoiding public places). Substituting these values into equation (43) yields the symptomatic contact rate: $\frac{80}{21} = \frac{7}{C_s} \Rightarrow C_s \approx 0.78$, and similarly, the asymptomatic contact rate: $\frac{80}{21} = \frac{26}{C_a} \Rightarrow C_a \approx 6.81$.. The same procedure was applied to estimate (C_s, C_a) for each of the remaining epidemic waves in our analysis.

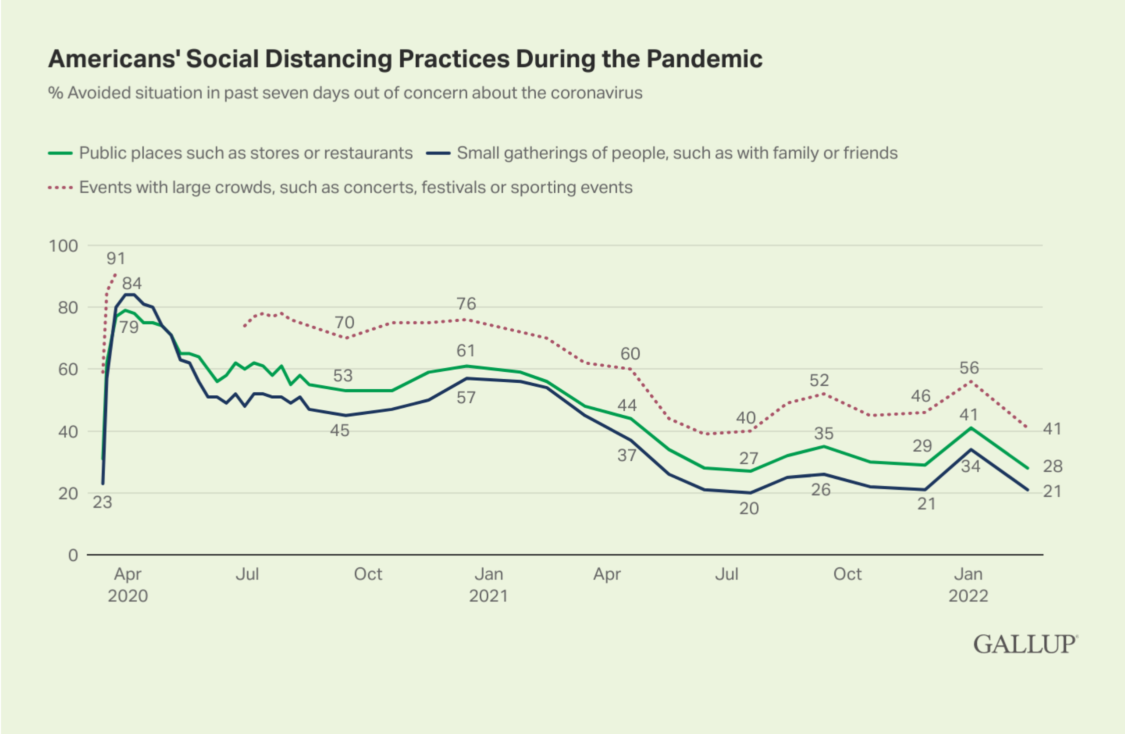

Figure S3: Percantages of Social Distance Practicing in USA. Source: GALLUP [1]

11 Compressions of Changes in Parameter Values

Figure S2 presents a visual comparison of the changes in model parameter values and basic reproduction numbers across successive COVID-19 waves.

Similarly, Figure S4 provides key insights into SARS-CoV-2 evolution and human interventions across different waves. Figure S4(a) shows vaccination-virulence dynamics, highlighting how vaccination rate (ξ_v) and disease-induced death rate (δ) evolved. During early waves (Wave 1 to Wave 3), δ remained stable as the original strain, D614G, and Alpha variants displayed moderate virulence. A sharp increase in δ during Wave 4 to Wave 5 reflects Omicron BA.1's heightened virulence, followed by a significant decline during Wave 5 to Wave 6, aligning with Omicron BA.2's reduced severity, supported by vaccination efforts (ξ_v). Figure S4(b) illustrates the asymptomatic transmission-virulence trade-off, with Omicron BA.1 showing a rise in asymptomatic transmission rate (β_a) and virulence, while Delta (Wave 3 to Wave 4) displayed moderate increases in β_a without major shifts in δ. The stabilization of β_a and decline in δ during Wave 5 to Wave 6 reflect Omicron BA.2's shift toward reduced virulence.

Figure S4(c) highlights the symptomatic transmission-virulence relationship, where Omicron BA.1 exhibited a sharp rise in symptomatic transmission rate (β_s) and virulence (δ) during Wave 4 to Wave 5.

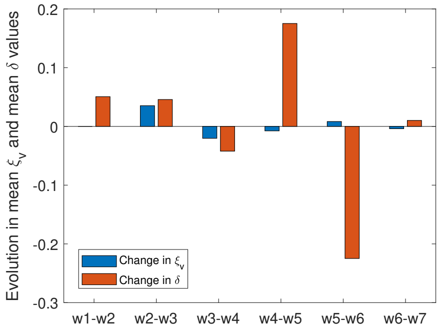

(a) Vaccination-Virulence Dynamics

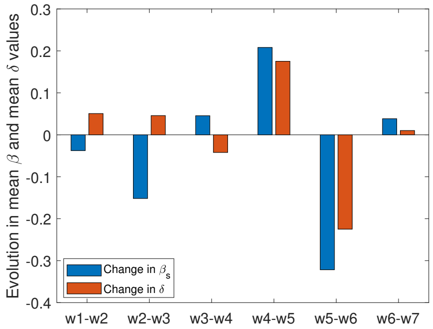

(b) Symptomatic Transmission-Virulence Trait

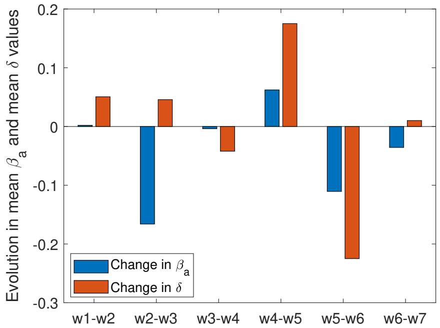

(c) Asymptomatic Transmission-Virulence Trait

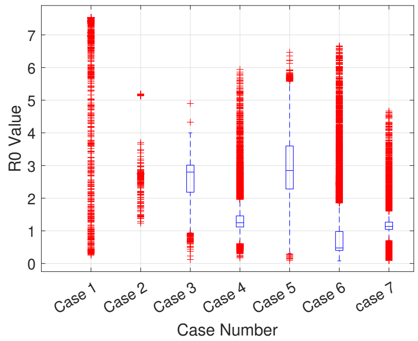

(d) Boxplot of Reproduction Number

Figure S4: *Visual analysis of key evolutionary and epidemiological traits across COVID-19 case transitions. **(a)** Dynamics of vaccination and virulence, showing changes in vaccination-induced immunity loss and virulence parameters. **(b)** Symptomatic transmission-virulence trade-off, illustrating how symptomatic transmission rates evolve in relation to virulence across transitions. **(c)** Asymptomatic transmission-virulence trade-off, highlighting shifts in asymptomatic transmission rates. **(d)** Boxplot of reproduction numbers (R_0) for each case, depicting the variability and trends in pathogen transmissibility over the seven epidemic waves.*

Earlier waves, including Delta, showed increased β_s without a corresponding rise in δ. The decline in both β_s and δ during Wave 5 to Wave 6 is consistent with Omicron BA.2's reduced severity. Figure S4(d) presents a boxplot of reproduction numbers (R_0), showing declines during early waves (Wave 2 to Wave 3) due to interventions like reduced contact rates and vaccination. A sharp rise in R_0 during Wave 5 reflects Omicron BA.1's dominance, while its decline in Wave 6 corresponds to Omicron BA.2's reduced virulence. Wave 7, dominated by XBB1.6, shows a moderate increase in R_0, indicating advanced immune escape.

12 Goodness of Model Fit

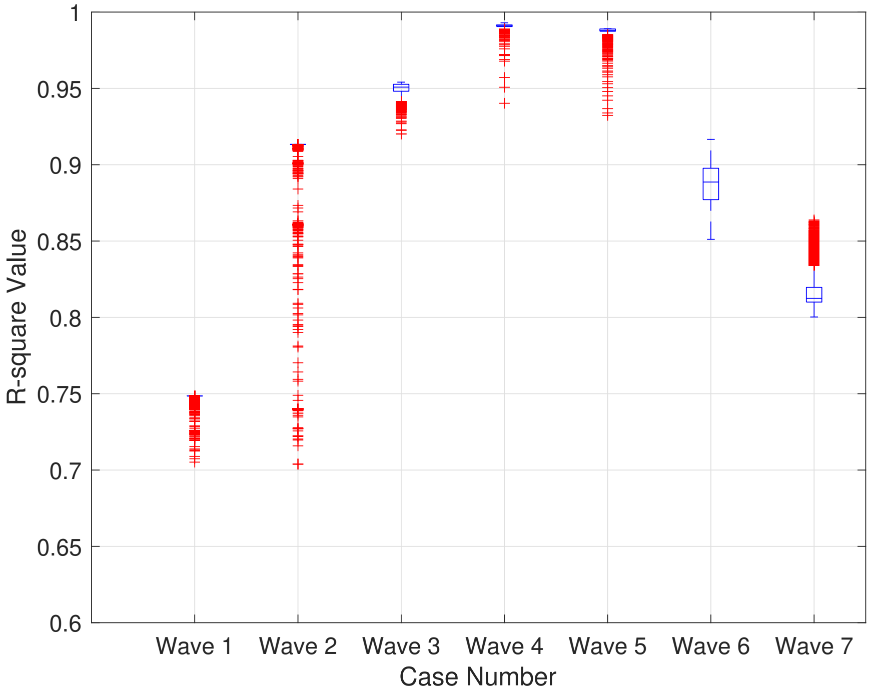

Figure S5: R-squared (R^2) values across seven consecutive COVID-19 waves

Figure S5 presents the goodness-of-fit of the model to the observed epidemic data. The consistently high R^2 values, all exceeding 0.7 and approaching 1 in several waves, demonstrate the model's ability to capture the underlying transmission dynamics across different epidemic phases. These results confirm that the model provides a reliable representation of COVID-19 dynamics, supporting its applicability for hypothesis testing and epidemiological forecasting.

References

[1] gallup. Usa social distance practices in pandemic. https://news.gallup.com/poll/390587/social-distancing-low-point-pandemic-anniversary.aspx.

[2] Chia-Yu Liu, Jessica Berlin, Moses C. Kiti, Emanuele Del Fava, Andre Grow, Emilio Zagheni, Alessia Melegaro, Samuel M. Jenness, Saad B. Omer, Ben Lopman, and Kenrad Nelson. Rapid review of social contact patterns during the COVID-19 pandemic. *Epidemiology*, 32(6):781–791, November 2021.
